# A Novel PhoP/PhoQ Regulation Pathway Modulates the Survival of Extraintestinal Pathogenic *Escherichia coli* in Macrophages

**DOI:** 10.3389/fimmu.2018.00788

**Published:** 2018-04-17

**Authors:** Xiangkai Zhuge, Yu Sun, Feng Xue, Fang Tang, Jianluan Ren, Dezhi Li, Juanfang Wang, Min Jiang, Jianjun Dai

**Affiliations:** ^1^MOE Joint International Research Laboratory of Animal Health and Food Safety, College of Veterinary Medicine, Nanjing Agricultural University, Nanjing, China; ^2^Center for Post-Doctoral Studies of Veterinary Medicine, College of Veterinary Medicine, Nanjing Agricultural University, Nanjing, China; ^3^Key Laboratory of Animal Bacteriology, Ministry of Agriculture, Nanjing Agricultural University, Nanjing, China

**Keywords:** extraintestinal pathogenic *Escherichia coli*, macrophages, PhoP/PhoQ, ColV plasmid, HlyF, autophagosome

## Abstract

The extraintestinal pathogenic *Escherichia coli* (ExPEC) is a typical facultative intracellular bacterial pathogen. Sensing the environmental stimuli and undertaking adaptive change are crucial for ExPEC to successfully colonize in specific extraintestinal niches. The previous studies show that pathogens exploit two-component systems (TCSs) in response to the host environments during its infection. The PhoP/PhoQ is a typical TCS which is ubiquitous in Gram-negative bacteria. However, there is an incompletely understanding about critical regulatory roles of PhoP/PhoQ in ExPEC pathogenesis. Conjugative ColV-related plasmids are responsible for ExPEC virulence, which is associated with ExPEC zoonotic risk. In this study, the molecular characteristics of HlyF, Mig-14 ortholog (Mig-14p), and OmpT variant (OmpTp) encoded by ColV plasmids were identified. Mig-14p and OmpTp played important roles in conferring ExPEC resistance to cationic antimicrobial peptides (CAMPs) during the infection. Moreover, HlyF and Mig-14p acted as intracellular survival factors to promote ExPEC resistance to macrophages killing. The *hlyF* and *Mig-14p* formed an operon in ExPEC ColV plasmid, and PhoP acted as a transcriptional activator of *hlyF* operon by directly binding to the P*_**hlyF**_* promoter. The acidic pH and CAMPs could additively stimulate ExPEC PhoQ/PhoP activities to upregulate the expression of HlyF and Mig-14p. Our studies revealed that the novel PhoP/PhoQ-HlyF signaling pathway directly upregulates the production of ExPEC outer membrane vesicles. Furthermore, our study first clarified that this PhoP/PhoQ-HlyF pathway was essential for ExPEC intracellular survival in macrophages. It was required to prevent the fusion of ExPEC-containing phagosomes with lysosomes. Moreover, PhoP/PhoQ-HlyF pathway facilitated the inhibition of the phagolysosomal acidification and disruption of the phagolysosomal membranes. In addition, this pathway might promote the formation of ExPEC-containing autophagosome during ExPEC replication in macrophages. Collectively, our studies suggested that PhoP/PhoQ system and CloV plasmids could facilitate ExPEC survival and replication within macrophages.

## Introduction

Extraintestinal pathogenic *Escherichia coli* (ExPEC) has the selective advantages over intestinal pathogenic *E. coli* (IPEC) to get access to extraintestinal niches, followed by efficient adaption/colonization in the host. ExPECs cause systemic disease among birds, humans, and mammals with typical extraintestinal pathology, including persistent bacteriuria in urinary tract infection, human septicemia or meningitis in newborns (1–4). The ExPECs were classified into four predominant phenotypes, including avian pathogenic *E. coli* (APEC), uropathogenic *E. coli* (UPEC), neonatal meningitis *E. coli* (NMEC), and septicemic *E. coli* (5). In recent years, ExPEC has been gradually accepted as a primary pathogen rather than the opportunistic pathogen (6–9). Compared with IPECs, ExPEC possesses certain-specific virulence/fitness factors to facilitate its extraintestinal infection. These virulence factors are involved in the adhesion, invasion, tolerance to and subversion of host immune defense (10–12). Interestingly, many studies confirm that APEC contaminates poultry meat or eggs, causing human extraintestinal diseases. The studies on animal models mimicking human ExPEC infection showed that APEC/ExPEC isolates originated from poultry can cause bacteremia, sepsis, urinary tract infection, and meningitis. More importantly, humans may be infected by these ExPEC isolates through consumption of unhygienic poultry food, adding another concern about poultry food safety (13, 14).

When ExPEC colonizes in urinary tract, respiratory and central nervous systems (the brain and meninges), it must evade the host innate immune defense, including both cellular components (e.g., macrophages) and immune factors (e.g., complement proteins) (15–18). ExPEC replicates in lung epithelial cells and then escapes from phagocytes clearance to enter the bloodstream. *E. coli* K1 can suppress macrophages clearance and acquire macrophages to pass through the blood–brain barrier using Trojan Horses strategy (19). More and more evidences confirm that ExPECs is a facultative intracellular pathogen (11, 12, 20), and persistence within macrophages is required for ExPEC dissemination. However, there is an incompletely understanding of intracellular survival mechanism in ExPEC pathogenesis.

Conjugative ColV-related plasmids are responsible for ExPEC virulence and have been widely isolated from APEC, UPEC, and NMEC (21). ColV-related virulence factors enhance ExPECs colonization and fitness during the infection (21). Moreover, the zoonotic risk of APEC seems to be associated with large ColV plasmids (21–23). The *hlyF* gene in ColV-related plasmids is an important epidemiology marker for highly virulent ExPEC, which was first discovered as a potential hemolysin protein in APEC (21, 24). A recent report clearly points out that HlyF is a virulence factor that can directly mediate the production of ExPEC outer membrane vesicles (OMVs) (23). The adjacent genes on each side of *hlyF* were putative Mig-14 ortholog and OmpT variant. Mig-14 is a *Salmonella* inner membrane protein can degrade cationic antimicrobial peptides (CAMPs), which is required for *Salmonella* survival/replication within macrophages and its persistent infection (25). CAMPs are cationic small peptides that display broad-spectrum antimicrobial effects on bacteria (26). OmpT locates in the outer membrane of *E. coli*, belonging to a class of highly homologous aspartyl proteases that can degrade host-derived AMPs. Several OmpT variants are present in pathogenic bacteria, including Pla in *Yersinia pestis*, SopA in *Shigella flexneri*, and PgtE in *Salmonella enterica* (27, 28).

Sensing the environmental stimuli and undertaking adaptive change are crucial for ExPEC pathogenesis. Pathogens exploit two-component systems (TCSs) in response to the host environments during their infection (29). The comparative genomic analysis shows that about 62 TCSs genes are conserved in *E. coli* genomes. However, there are little studies on the roles of TCSs in ExPEC pathogenesis (29). The PhoP/PhoQ, a typical TCS, is broadly conserved in Gram-negative bacteria; it can sense host intracellular signals and regulate bacterial adaptive lifestyle change during its infection (30, 31). Over the past decade, the function of PhoP/PhoQ is widely studied in various pathogens. PhoQ has a periplasmic domain to sense and transfer signals to its cytoplasmic histidine kinase domain, which governs the phosphorylation level of the response regulator PhoP. The PhoP can directly bind the promoters of the regulatory genes which have the consensus binding motif “TGTTTA(N5)-TGTTTA” in *E. coli* and *S. enterica* (32, 33). However, there is an incompletely understanding about the regulatory roles of PhoP/PhoQ in ExPEC pathogenesis.

In this study, the roles of ColV plasmid-encoded virulence factors HlyF, Mig-14p, and OmpTp were established. We identified a novel PhoP/PhoQ-HlyF signaling pathway that played the important roles in ExPEC survival and replication within macrophages.

## Materials and Methods

### Strains and Plasmids Construction

The strains, plasmids, and the PCR primers used in the studies were described in Tables S1 and S2 in Supplementary Material. The highly virulent FY26 (O2:K1; ST95; ECOR B2; isolated from chicken), is a typical model used to study the ExPEC pathogenesis. The *hlyF*-, *Mig-14p*-, *OmpTp*-, and *phoP-*deficient FY26 mutants, and others deletion mutants were constructed by Red recombinase method previously described (15). The *hlyF*:*lacZ-zeo* fusion transcriptional reporter in FY26Δ*lacI-Z* was constructed using Red recombinase system according to the previous study by Vigil (11). Briefly, two targeted fragments were amplified: the *lacZ* fragment containing the homology region to 5′ end of *hlyF* sequence (starting at the 30 bp site after *hlyF* start codon), and the other fragment containing the kanamycin resistance and the homology region of 3′ end of *hlyF*. The fusion PCR was performed to integrate the two fragments. *hlyF*:*lacZ-zeo* fusion reporter was constructed in FY26Δ*lacI-Z* by Red recombinase system. To construct the complemented plasmids for stable expression of HlyF, Mig-14p, OmpTp, and PhoP, a medium-copy plasmid pSTV28 (pACYC184 origin, TaKaRa) was used as the carrier (15). The *hlyF*/*Mig-14p* operon, *OmpTp*, and *phoP* genes (containing the predicted promoters) were amplified and ligated into pSTV28, and the complemented plasmids were electroporated into the corresponding FY26 mutants, respectively.

To construct the plasmids overexpressing GST:HlyF and MBP:Mig-14p fusion proteins, the *hlyF* and *Mig-14p* genes were cloned into expression plasmids pCold-GST and pCold-malE, respectively, which were constructed by ligating GST or MBP coding gene into the pCold I (TaKaRa) using the single *NdeI* site. To construct the plasmids overexpressing OmpTp and PhoP fusion proteins, the *OmpTp* and *phoP* genes were cloned into expression plasmid pET-28a (Novagen). The plasmids were transformed into *E. coli* BL21 (DE3). GST:HlyF, MBP:Mig-14p, OmpTp, and PhoP proteins were purified by a HisTrap high-performance column (GE Healthcare, Shanghai, China) (15).

### Cell Culture

HD11 cells (chicken macrophage-like cell line) were derived from chicken bone marrow macrophages by transforming the avian leukemia virus (34). HD11 macrophages were kindly provided by Shaohui Wang (Shanghai Veterinary Research Institute, Chinese Academy of Agricultural Sciences) and cultured as the previously described (15, 35). HD11 cells were cultured in RPMI 1640 media (Gibco) (at a humidified incubator for 41.5°C and 5% CO_2_), supplemented with 10% heat-inactivated fetal bovine serum (FBS), 2 mM l-glutamine (Gibco), 1 mM of sodium pyruvate 0.1 mM of NEAA, and 10 mM HEPES. U937 cells (human monocytic lymphoid cell line) was originally obtained from the ATCC and cultured in RPMI medium (Gibco) as the previously described (36). U937 cells were maintained in RPMI medium (at a humidified incubator for 37.5°C and 5% CO_2_), supplemented with 10% FBS (Gibco) and 2 mM l-glutamine. To obtain a macrophage-like phenotype, U937 cells were differentiated with 25 ng/ml phorbol 12-myristate 13-acetate for 24 h.

### RNA Isolation, Reverse Transcription PCR, and Quantitative Real-Time RT-PCR (qRT-PCR)

The total RNA of wild-type FY26, mutants, and complemented strains [cultured in LB (pH 7.4) under mid-logarithmic phase] were extracted using TRIzol^®^ Reagent (Invitrogen); DNase I (TaKara) was added to remove the genomic DNA according to the manufacturer’s instruction. The total RNA isolated from FY26, FY26Δ*phoQ*, or FY26C*phoQ* [cultured in M9 media with 10 mM Mg^2+^ at pH 7.5/pH 5.0 (acidic pH) or with CAMPs] was also extracted using TRIzol^®^ Reagent (Invitrogen). The RNA concentration and quality were determined using 2100 Bioanalyser (Agilent) and quantified using the ND-2000 (NanoDrop Technologies). The total RNA was treated with DNase for 1 h, and then PCR was conducted using the treated RNA as the templates to detect the DNA contamination. The total RNA from the infected HD11 or U937 was extracted according to the previous study (15).

For the co-transcription test, the treated bacteria RNA was reverse transcribed into cDNA using a SuperScript II reverse transcriptase kit (Invitrogen). The qPCR was performed to assess the co-transcription of intergenic regions using the primers that spanned the 3′ end of one gene to the 5′ end of the adjacent genes (Table S2 in Supplementary Material) according to the previous study (15). The RNA samples without reverse transcription were used as a negative control to rule out the DNA contamination.

The real-time PCR was conducted (10), and the primers for qRT-PCR were shown in Table S2 in Supplementary Material. The transcription level of the housekeeping gene *dnaE* was used as a reference to determine the expression level of the target genes with the ΔΔ*C_T_* method (15). qRT-PCR was conducted using the AceQ qPCR SYBR Green Master Mix (Vazyme, Nanjing) according to the manufacture’s instruction (10).

### Electrophoretic Mobility Shift Assays (EMSAs)

To determine the binding of PhoP to DNA probe of *hlyF* promoter, EMSAs were conducted using the commercialized EMSA kit (Invitrogen, California) according to the manufacturer’s protocol (15). To obtain phosphorylated PhoP, purified PhoP was phosphorylated with acetylphosphate (Sigma) according to the previous study (37). PhoP (10 µM) was incubated in 100 µl of phosphorylation buffer (20 mM HEPES, pH 7.5, 100 mM NaCl, and 5 mM MgCl_2_) containing 20 mM acetylphosphate for 2 h at 37°C. To obtain the DNA probes, the P*_hlyF_* DNA fragment (200 bp in size, starting from upstream position -170 bp to downstream position +30 bp relative to the position of the translational start codon) and the negative control DNA fragment (200 bp in size for *hlyF* coding region) were amplified with the corresponding primers (Table S2 in Supplementary Material). The PCR products were purified using the agarose gel DNA fragment recovery kit (TaKaRa). The mutated P*_hlyF_* DNA with nucleotide deletion at promoter P*_hlyF_* position nt −68 to −63 (PhoP box mutant) was prepared by fusion PCR. EMSAs were conducted by adding increasing amounts of non-phosphorylated or phosphorylated PhoP protein (0–2.0 µM) to the DNA probe (50 ng) in the premixed EMSA binding buffer. The reactions were carried out for 45 min at room temperature. Then the samples were injected in the 6% polyacrylamide gels, and the electrophoresis was performed in 0.5× TBE buffer at 200 V for 30 min. The gels were photographed using the gel imaging system (Bio-Rad) after incubation with 1× SYBR Gold nucleic acid staining solution in 0.5× TBE buffer for 30 min.

### β-Galactosidase Assays

The bacteria harboring the *hlyF*:*lacZ* fusion transcriptional element were cultured overnight in LB medium. The overnight cultures were diluted 1:100 in LB medium and grown to mid-log phase (OD_600_ 0.6–0.8) at 37°C. The culture was centrifuged, and the bacteria were resuspended and diluted 1:10 in Z buffer (pH 7.0; 60 mM Na_2_HPO_4_·7H_2_O, 40 mM NaH_2_PO_4_, 1 M KCl, 1 mM MgSO4, 50 mM β-mercaptoethanol). The Miller assay to detect β-galactosidase activity was conducted using *ortho*-nitrophenyl-β-galactoside as the substrate (15). The tests for β-galactosidase activity were repeated three times.

### CAMP Sensitivity Assays

The sensitivities of wild-type FY26, derived mutants, and complemented strains to AMPs (LL-37 and HBD2) were determined (25, 38). The AMPs were twofold serially diluted (1.92 mg/ml to 0.12 µg/ml) in PBS containing 0.01% BSA. The 10 µl diluted AMPs were added to the 90 µl LB medium containing about 1.0 × 10^5^ CFU for the tested strain. The bacteria were grown overnight at 37°C, and the cultured wells were visually inspected for the inhibition of bacteria growth. Values shown in Table S3 in Supplementary Material represented at least three independent MICs tests for eight replicates. Furthermore, we followed the research methodology of Murase et al. to transform the recombinant plasmids (pSTV28-hlyF, pSTV28-hlyF/Mig-14p, and pSTV28-OmpTp) in non-hlyF strain RS218 (23). RS218 is a well characterized prototypic NMEC O18:K1 strain without ColV plasmids (39, 40). The comparative genomic analysis of ExPEC O18:K1 strains demonstrated very close genetic overlap/similarities, as well as indistinguishable virulence genes features with a class of O1:K1 and O2:K1 isolates (3, 4, 41). Therefore, the complemented variants that overexpressed the HlyF, Mig-14p and OmpTp in RS218 were constructed to determine their abilities to resistance to CAMPs.

Time-kill tests using CAMPs (LL-37 and HBD2) were performed according to the previous studies with some modifications (25, 38). The bacterial cells grown to mid-log phase in LB (pH 7.4) were harvested by centrifugation and resuspended in LB (pH 7.4) to 1.0 × 10^9^ CFU/ml. The bacterial cells were diluted 1:10 into LB medium containing LL-37 (100 µg/ml) or HBD2 (60 µg/ml). The AMPs treated cells were inoculated at 37°C without shaking for 2 h. The cells samples were serially diluted and plated on LB agar plates for counting. Time-kill assays were carried out in triplicate. Percent survival for each tested strain was measured relative to wild-type FY26.

### Bacterial Fractionation and Components Separation

Bacterial fractionation was conducted according to the previously described method (10, 11). Briefly, the cultured bacteria in 100 ml medium were collected by centrifugation and washed twice with PBS, and the bacteria were resuspended in 5 ml PBS buffer containing 100 mM NaCl. The supernatant was concentrated by Millipore centrifugal filter unit (10 kDa cutoff), and the proteins in the supernatant were subjected to ammonium sulfate precipitation. The pellets were resuspened in PBS and dialyzed twice in PBS at 4°C. The cells were lysed by sonication and centrifuged (10,000 × *g*) to remove the cell debris. The collected lysate was centrifuged at 300,000 × *g* for 2 h at 4°C. The precipitation (bacterial membrane components) and the supernatant (soluble cytoplasmic components) were subjected to ultracentrifugation. The supernatant containing soluble cytoplasmic proteins were concentrated by Millipore centrifugal filter unit (10 kDa cutoff), and the cytoplasmic proteins were subjected to ammonium sulfate precipitation. The pellets were resuspened in PBS and washed twice. Under room temperature, the precipitation was incubated with 1% Triton X-100 for 30 min. The membrane fractions were separated by ultracentrifugation at 150,000 × *g* for 1 h, and the supernatant (containing the inner membrane proteins) and the pellets (the outer membrane proteins) were collected and stored at 70°C.

### Western Blot Analysis

To prepare the mouse anti-HlyF, anti-Mig-14p, and anti-OmpTp serums, 8-week-old imprinting control region mice were subcutaneously immunized with the fusion proteins (10). The same volume of fusion proteins (150 µg) was mixed with Montanide ISA 206 adjuvant (SEPPIC, Lyon, France) to immunize the mouse. The anti-serum was collected after the third immunization. In addition, a polyclonal rabbit anti-APEC serum was prepared in the previous study (15).

The expression of HlyF, Mig-14p, and OmpTp in wild-type FY26, the mutants, and corresponding complemented strains were analyzed by Western blot. The bacteria were subjected to SDS-PAGE electrophoresis and transferred to a polyvinylidene difluoride (PVDF) membrane with a semidry blotting apparatus. The PVDF membrane was blocked by incubation overnight in TBST containing 5% non-fat milk at 4°C. The immunoblotting assays were conducted with polyclonal anti-serums (primary antibodies) corresponding to fusion proteins (GST:HlyF, MBP:Mig-14p, and OmpTp). For α-sigma 70 detection, a commercial anti-sigma 70 antibody (Abcam, ab12088) was used at a 1:1,000 dilution in TBST buffer. The horseradish peroxidase-conjugated anti-mouse IgG or anti-rabbit IgG were used as the secondary antibody. The enhanced chemiluminescence (Vazyme Biotech Co., Ltd.) was used for immunoblotting detection.

To analyze subcellular localization of HlyF, Mig-14p, and OmpTp in wild-type FY26, the four extracted subcellular components (OM, outer membrane proteins; Sup, concentrated culture supernatant; IM, inner membrane proteins; Cyt, cytoplasmic component) were subjected to 12% polyacrylamide gels and then transferred to a PVDF membrane. The outer membrane protein groEL and cytoplasmic protein α-Sigma70 were used as the positive control. For groEL detection, a commercial anti-groEL antibody (Abcam) was used at a 1:1,000 dilution in TBST buffer. The enhanced chemiluminescence (Vazyme Biotech Co., Ltd.) was used for immunoblotting detection.

To analyze LC3 and p62 in FY26-infected HD11 cells by western blot, HD11 cells were infected with FY26 at a multiplicity of infection (MOI) of 5. At 1, 2, 4, 6, or 8 h post-infection (hpi), the cells were washed twice with PBS and then scraped from 6-well plate. Furthermore, HD11 cells were treated with rapamycin for 4 h as a positive control of autophagy induction, and incubated with FBS-free RPMI 1640 media as the negative control. The cells were lysed by incubation with cell lysis buffer (50 mM Tris–HCl, 2 mM EDTA, 0.1% SDS, 150 mM NaCl, 1% Triton X-100, 5 mM sodium orthovanadate, 0.1 mM PMSF, and pH 7.4) containing a protease inhibitor cocktail (Roche). The supernatant of cell lysates was collected by centrifugation (12,000 × *g*) for 20 min at 4°C. The protein concentration of the supernatant was determined using the BCA assay (Thermo). SDS-PAGE was performed with equal amounts of cell proteins. The western blot was performed by using anti-LC3 antibody (Abcam, ab63817), anti-p62 antibody (Abcam, ab101266), and anti-β-actin antibody (Abcam, ab8227). Densitometry analysis was performed to determine the relative quantification. The data for the ratio of LC3-II and p62 to β-actin were acquired from three independent experiments.

### Intracellular Survival Assay

The intracellular survival assays were performed according to the previous study (15). HD11 cells were infected with FY26 variants for an MOI of 10. After 1 h of infection, infected cells were treated with gentamicin for 1 h to kill the extracellular bacteria. The bacteria in HD11 cells after treated with gentamicin at different time points (2, 3, 5, 7, and 15 h) were measured by plate counting. The internalized bacteria at 2 hpi were used as the initial number of intracellular bacteria for data calculation. Intracellular survival level was calculated as change (*n*-fold) in bacterial number at a given time point relative to initially internalized bacteria. The intracellular survival assays were performed in triplicate.

### Immunofluorescence Microscopy

Immunofluorescence analysis for Mig-14p localization was performed according to the previously study with some modifications (10, 42). Briefly, bacteria cells were harvested by centrifugation and then washed twice with PBS. The cells were fixed in 2.67% paraformaldehyde and 0.01% glutaraldehyde for 15 min and then washed twice with GTE buffer (50 mM glucose, 25 mM Tris–HCl, 10 mM EDTA, and pH 8.0). The cells were resuspended in GTE, and incubated overnight at 4°C. The cells were permeabilized by incubation with 10 mg/ml of lysozyme and 5 mM EDTA for 30 min at room temperature, and then washed twice and blocked in 0.5% BSA for 15 min. The bacteria were treated with anti-Mig-14p serum (1:500 diluted in PBS containing 1% BSA) at 37°C for 2 h, and then washed three times. The bacteria were next incubated with goat anti-mouse IgG-FITC (1:500 dilution; EarthOx) for 1 h at 37°C and washed three times. The bacteria membrane was stained with 10 µM FM4-64 (Molecular Probes, Invitrogen) (43), and DNA was stained with 2 µg/ml DAPI (Sigma-Aldrich). The bacterial suspensions (10 µl) were visualized and photographed using a Zeiss LSM-510 META confocal laser scanning microscope.

To visualize and quantify the intracellular bacteria, immunofluorescent imaging assays were performed (15, 36). Briefly, the infected HD11 cells at the MOI of 5 were washed with PBS and fixed in 3% paraformaldehyde, and then treated with 0.1% Triton X-100 in PBS for 3 min. After blocked with 5% BSA in PBS, cells were incubated for 2 h with the different diluted primary antibodies, including the commercial rabbit polyclonal anti-Ubiquitin (Ub) antibody (Abcam, EPR8589), anti-LC3B antibody (Abcam, ab63817), and anti-LAMP1 antibody (Abcam, ab24170). The rabbit polyclonal anti-galectin 8 (Gal8) antibody was prepared from the rabbit serum immunized with purified recombinant chicken Gal8/LGALS8 (*Gallus gallus*, reference sequence: XM_015284119.1).

The infected cells were fixed in 2.67% paraformaldehyde and 0.01% glutaraldehyde for 15 min, and blocked in 2.5% BSA for 1 h. Then washed cells were treated with TRITC goat anti-rabbit IgG (EarthOx, San Francisco) at 37°C for 1 h. The washed cells were incubated for 1 h with mouse polyclonal anti-ExPEC antibody and then treated with goat anti-mouse Alexa Fluor 488-conjugated IgG. The cells were next incubated with DAPI at 37°C for 30 min. Samples were washed three times and observed using a Zeiss LSM-510 META confocal laser scanning microscope. For LysoTracker labeling, cells were incubated with LysoTracker (red DND-99; Invitrogen) at a final concentration of 75 nM for 1 h before ExPEC infection. The number of intracellular bacteria could be directly counted from immunofluorescent imaging. Data for quantification of the colocalization rates represented the results of more than 100 infected HD11 cells in at least three independent tests.

### Isolation of OMVs

The bacterial OMVs were isolated according to previous studies with slight modifications (23, 44, 45). Briefly, the bacteria were cultured in 500 ml of LB medium for 16 h (37°C, 180 rpm). The cultured supernatant was collected by centrifugation (10,000 × *g*) to remove the bacteria. To remove the bacteria completely, the supernatant was further filtered twice by a 0.22 µm sterile filter. OMVs were isolated from the filtered supernatants by ultracentrifugation (200,000 × *g*, 2 h, 4°C) in a 45 Ti rotor (Beckman). The obtained OMVs precipitation was washed once with PBS and subjected to ultracentrifugation again. The OMVs pellets were resuspended in 50 µl TE buffer and stored at −80°C. The isolated OMVs were visualized by transmission electron microscopy (TEM) under 100,000× original magnification according to the previous described (23). Quantification of the OMVs was performed from more than 10 images (high-power field) for each strain using the ImageJ software. The average number of OMVs per field was quantified from at least four independent experiments.

## Results

### Molecular Characterization of HlyF, Mig-14 Ortholog (Mig-14p), and OmpT Variant (OmpTp) Encoded by ColV Plasmids

**Figure 1:**
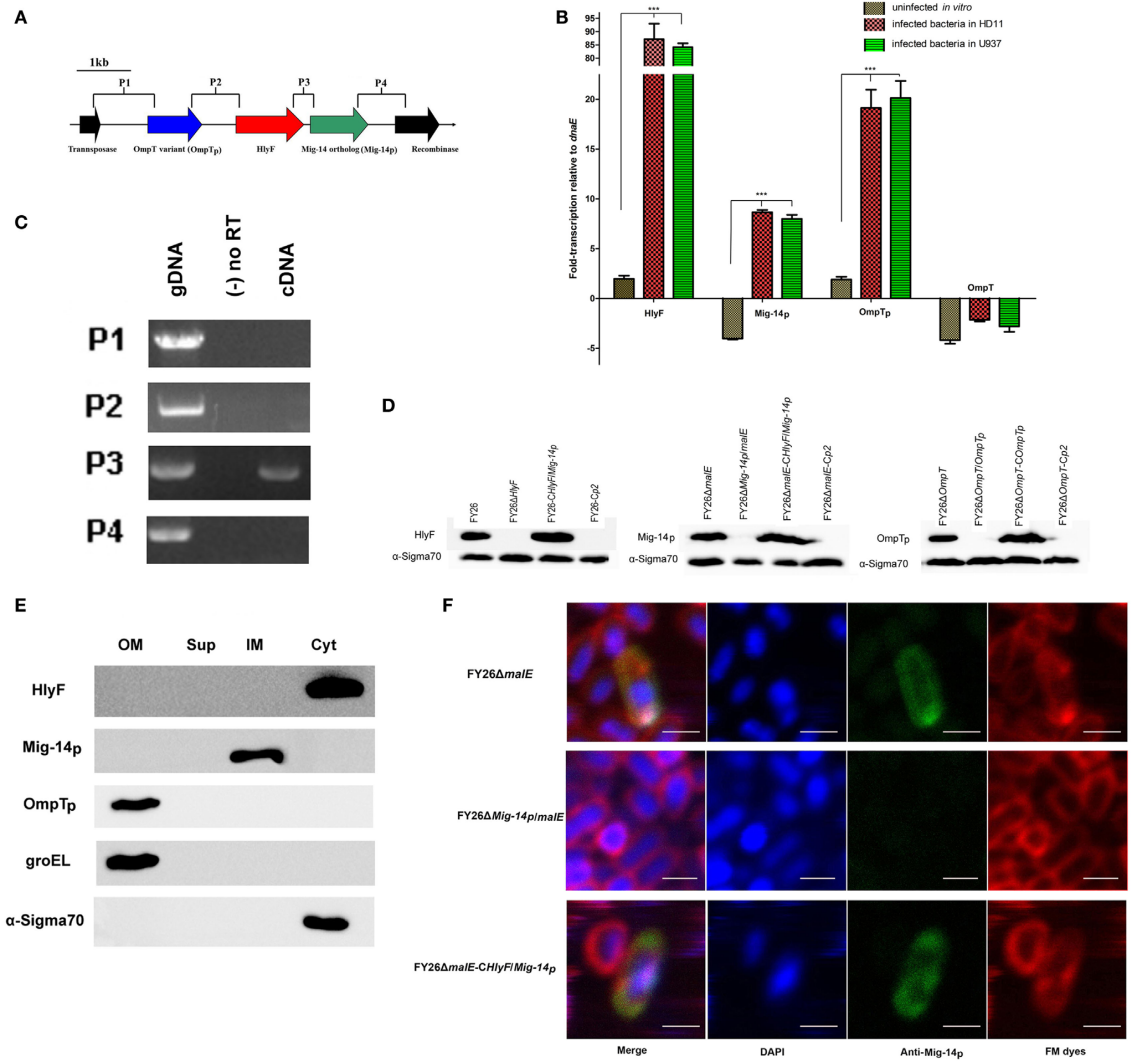
Molecular characterization and subcellular localization of HlyF, Mig-14p, and OmpTp encoded by ColV plasmids in extraintestinal pathogenic *Escherichia coli* (ExPEC) strain FY26. **(A)** Chimeric feature and genetic context of *hlyF, Mig-14p*, and *OmpTp* located in ColV plasmids. The arrows and its direction indicated the gene size and transcription direction for these genes. **(B)** The transcription levels of *hlyF, Mig-14p, OmpTp*, and *ompT* (negative control) in strain FY26 under different conditions by quantitative real-time RT-PCR (qRT-PCR). The transcriptional differences of these genes during FY26 infecting HD11 and U937 cells were determined relative to that in routine culture condition (uninfected *in vitro*). The qRT-PCR data (relative to housekeeping gene *dnaE*) from three independent experiments were used to identify the transcriptional differences (fold change), and statistically significant differences were determined using one-way ANOVA analysis (***P < 0.01). **(C)** The co-transcription test for intergenic regions of *hlyF, Mig-14p, OmpTp*, and adjacent genes by PCR. Total RNA of FY26 was reversely transcribed to cDNA (PCR amplification templates). The negative control was the non-reverse transcriptional RNA. **(D)** Western blot analysis for the expression of HlyF, Mig-14p, and OmpTp in wild-type FY26, the mutants, and corresponding complemented strains. The immunoblotting assays were conducted with polyclonal anti-serums corresponding to fusion proteins (GST:HlyF, MBP:Mig-14p, and OmpTp), and detecting α-Sigma 70 expression acted as a reference. **(E)** Western blot analysis for subcellular localization of HlyF, Mig-14p, and OmpTp in wild-type FY26. Four FY26 subcellular components (OM, outer membrane proteins; Sup, concentrated culture supernatant; IM, inner membrane proteins; Cyt, cytoplasmic component) were separated and used to immunoblotting assays. The outer membrane protein groEL and cytoplasmic protein α-Sigma70 acted as the positive control. **(F)** Immunofluorescent detection of Mig-14p protein in ExPEC. FY26Δ*malE*, FY26Δ*Mig-14p*/*malE*, and FY26Δ*malE*-C*HlyF*/*Mig-14p* for immunofluorescence detecting were fixed and probed with anti-MBP:Mig-14p serum and then labeled with fluorescent secondary antibodies conjugated to FITC. DAPI was used to label bacteria nucleic acid, and plasma membrane was stained by FM dyes. Scale bars = 1 μm.

To date, few studies have identified the molecular pathogenic mechanism of plasmid-encoding virulence factors from ExPEC isolates, particularly the ColV-related plasmids, which are ubiquitously distributed among ExPEC and responsible for the capabilities of ExPEC colonization and fitness during its infection. The *hlyF* and the adjacent virulence genes, encoding putative Mig-14 ortholog and OmpT variant, are conserved in ColV-related plasmids (Figure 1A). The sequence alignment showed ColV plasmid-encoded Mig-14 ortholog (Mig-14p) shared about 57% identity with *Salmonella* Mig-14; while OmpT variant (OmpTp) shares approximately 73% homology to *E. coli* chromosome-encoding OmpT (Figure S1 in Supplementary Material). qRT-PCR results showed that the *in vitro* transcriptional levels of *hlyF, Mig-14p*, and *OmpTp* were close to that of ExPEC housekeeping gene *dnaE* under routine condition. Furthermore, RNA levels of *hlyF, Mig-14p*, and *OmpTp* were significantly enhanced about 44.5-, 34.9-, and 10.1-fold in FY26-infected HD11 macrophages relative to those under routine condition, and similar results were detected in FY26-infected U937 cells (Figure 1B). Moreover, co-transcription test for intergenic regions was conducted to evaluate whether *hlyF, Mig-14p*, and *OmpTp* formed one operon. As shown in Figure 1C, the transcription between *hlyF* and *Mig-14p* could be detected, but not between *OmpTp* and *hlyF*, suggesting *hlyF* and *Mig-14p* belonged to one operon, whereas *OmpTp* was a single transcriptional unit.

For the expression of OmpTp, we referred to the relevant reports and selected the expression vector pET-28a for a small fusion protein tag (28, 46). However, HlyF and Mig-14p proteins by plasmid pET-28a or pET-32a were not successfully expressed. Then, we select the more hydrophilic labels MBP or GST to express the HlyF and Mig-14p fusion proteins (23). HlyF fusion protein was successfully expressed by plasmids pCold-GST and pCold-malE. And Mig-14p fusion protein could be expressed by the plasmid pCold-malE. The fusion proteins GST:HlyF, MBP:Mig-14p, and OmpTp were overexpressed and purified (Figure S2 in Supplementary Material), and the corresponding anti-serums for these fusion proteins were prepared. The deletion mutants of *hlyF, Mig-14p, OmpTp*, or other genes were constructed on wild-type FY26 (Table S1 in Supplementary Material). Subsequently, the complemented strains were constructed by transformation of plasmids (pSTV28-hlyF, pSTV28-hlyF/Mig-14p, and pSTV28-OmpTp) to the corresponding mutants (Table S1 in Supplementary Material). The expression of HlyF, Mig-14p, and OmpTp was detected by immunoblotting with the corresponding anti-serums. As shown in Figure 1D, the expression of HlyF, Mig-14p, and OmpTp could be detected in strain FY26 and complemented strains, but not in the corresponding mutant strains. The previous studies demonstrated that ExPEC HlyF is a cytoplasmic protein, and OmpT is located in ExPEC outer membrane (23, 28). As shown in Figure 1E, our immunoblotting results were consistent with these studies, showing HlyF is located in the cytoplasm of wild-type FY26, and OmpT is an outer membrane protein of wild-type FY26.

Mig-14 is a *Salmonella* inner membrane protein (25), and immunoblotting result revealed that Mig-14p was detected only in the inner membrane fraction of FY26 (Figure 1E). The immunofluorescent imaging of subcellular localization further showed that Mig-14p in FY26 and the complemented strain colocalized with the plasma membrane (staining by FM dyes), and deletion of Mig-14p abolished the colocalization (Figure 1F). The immunoblotting and fluorescent results identified that the putative Mig-14p acted as an inner membrane-related protein in ExPEC, similar to *Salmonella* Mig-14.

### PhoP/PhoQ Regulating the Transcriptional Expression of HlyF and Mig-14p

**Figure 2:**
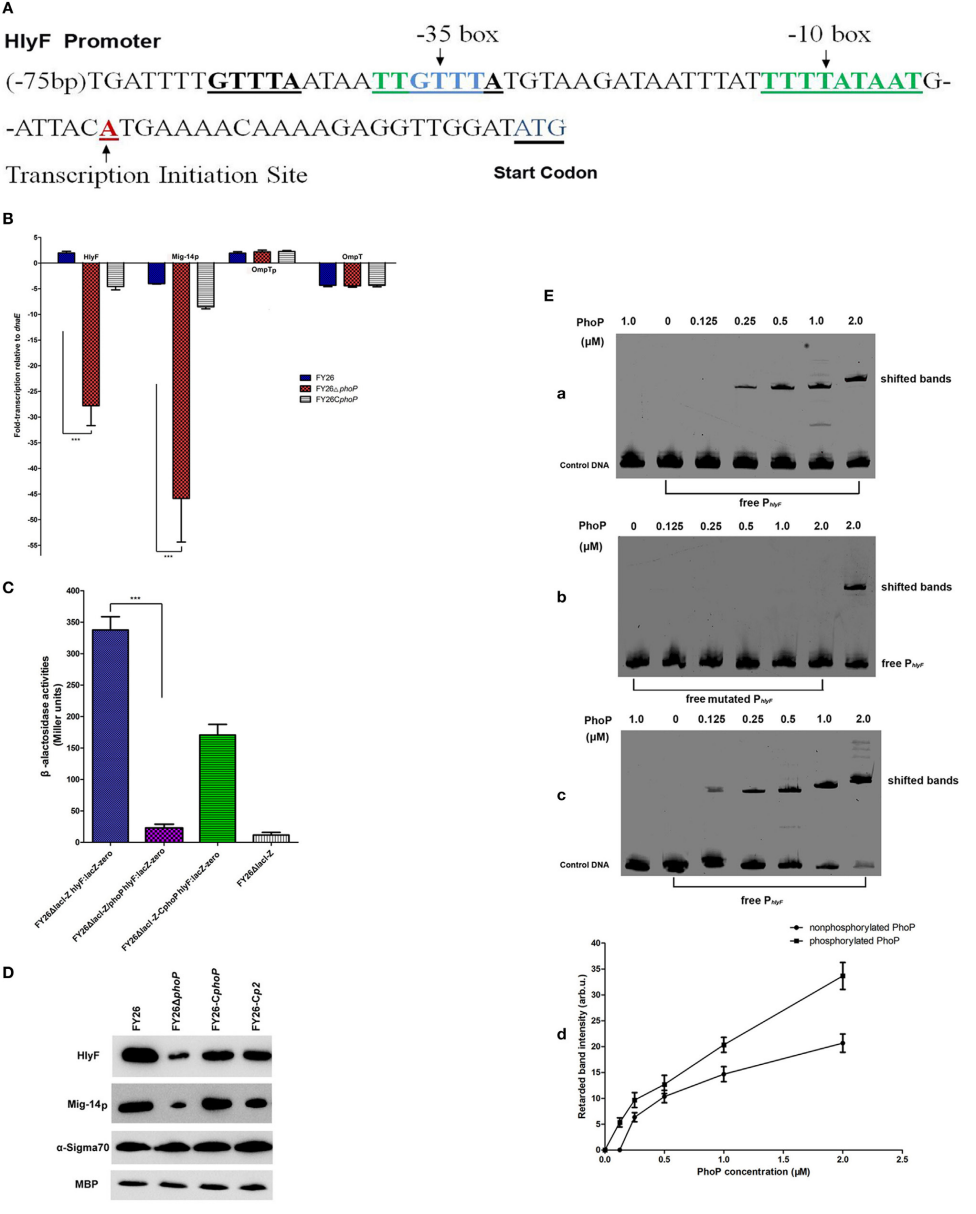

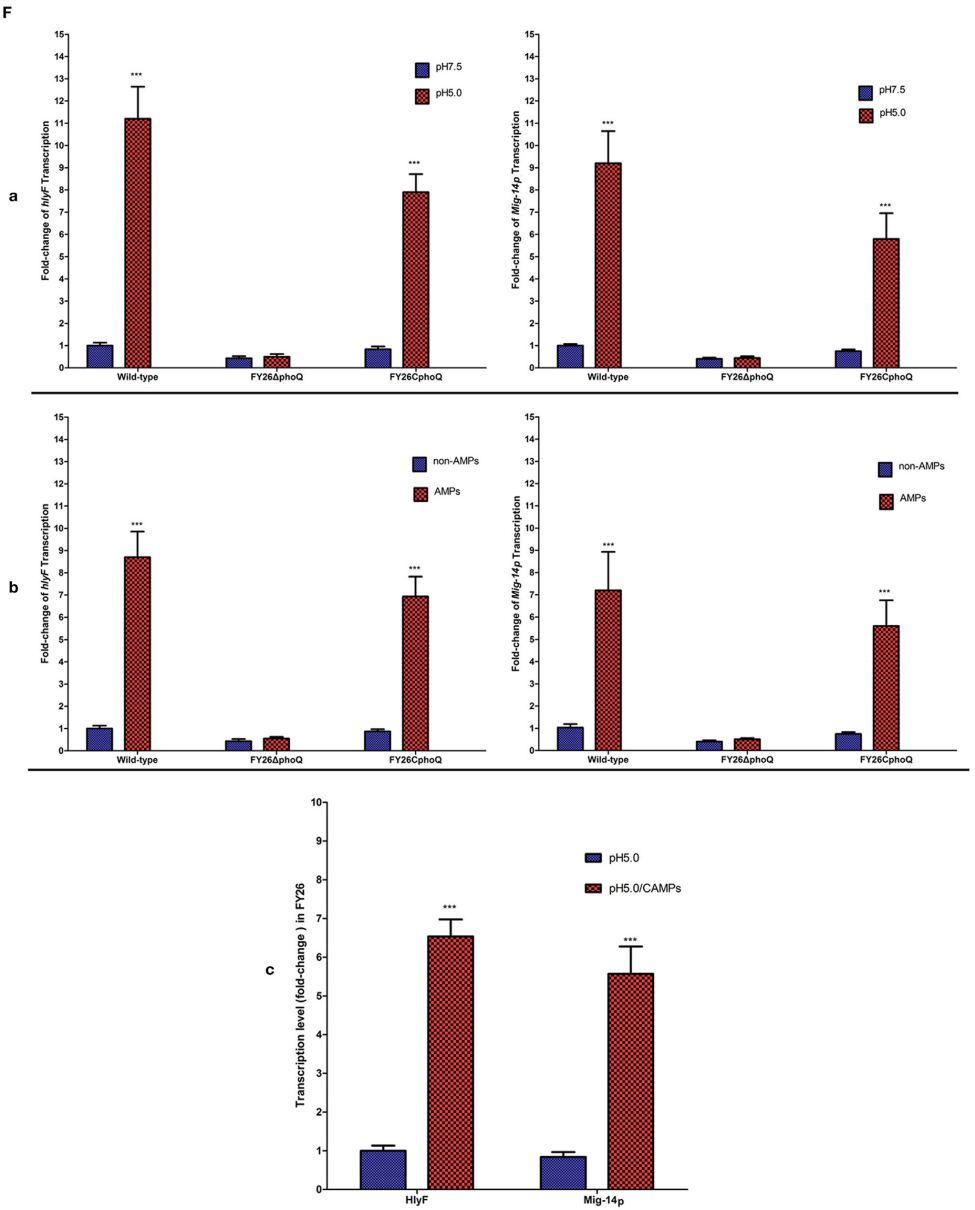
Molecular identification of PhoP/PhoQ regulating the transcriptional expression of HlyF and Mig-14p. **(A)** Schematic illustration of transcription promoter regions of *hlyF* and *Mig-14p* operon. The PhoP-binding motifs, −10/−35 consensus boxes, transcription initiation site and *hlyF* start codon were marked by underline. **(B)** Quantification of the transcription levels for *hlyF, Mig-14p, OmpTp*, and *ompT* among wild-type FY26, the *phoP* mutant, and complemented strain by quantitative real-time RT-PCR (qRT-PCR). The qRT-PCR data (relative to housekeeping gene *dnaE*) from three independent experiments were used to identify the transcriptional differences (fold change), and statistically significant differences were determined using one-way ANOVA analysis (***P < 0.01). **(C)** The β-galactosidase activities among FY26 variants, carrying *hlyF*:*lacZ* transcriptional fusion, were determined by the Miller assay. The *hlyF* transcriptional levels among FY26 variants were indirectly evaluated by measuring β-galactosidase activities. Data acquired from at least four independent experiments performed in triplicate, and the mean values ± SEs were shown. The statistically significant differences were determined using one-way ANOVA analysis (***P < 0.01). **(D)** Western blot analysis for HlyF and Mig-14p expression in wild-type FY26, the *phoP* mutant, and complemented strains. The immunoblotting assays were conducted with polyclonal anti-serums corresponding to fusion proteins (GST:HlyF and MBP:Mig-14p,). Detecting the expression of α-Sigma70 and MBP acted as references. **(E)** Characterization of interaction of PhoP with promoter P*_hlyF_* DNA by EMSA. [**(E)**, a] The purified non-phosphorylated PhoP could bind and shift the promoter P*_hlyF_* DNA. Non-radioactive EMSA was used to detect the band shift of promoter P*_hlyF_* DNA. The EMSA probes for P*_hlyF_* DNA fragment (200 bp) and the negative control (200 bp) for OmpTp promoter were amplified by PCR. EMSAs were performed by adding increasing amounts of non-phosphorylated PhoP in binding reactions, as described in Experimental Procedures. [**(E)**, b] EMSA showing specificity of PhoP binding to promoter P*_hlyF_* DNA. The mutated P*_hlyF_* DNA with nucleotide deletion at promoter P*_hlyF_* position nt −68 to −63 (PhoP-binding site) was amplified by PCR. EMSAs were performed to determine the binding affinity between P*_hlyF_* DNA and the mutated P*_hlyF_*. [**(E)**, c] Effect of PhoP phosphorylation on the P*_hlyF_* DNA binding affinity. The phosphorylated PhoP with different concentrations was added in the binding reactions. [**(E)**, d] The intensity of shifted P*_hlyF_* DNA bands between non-phosphorylated PhoP (a) and phosphorylated PhoP (c) was determined and plotted with PhoP protein concentration. **(F)** The acidic pH and CAMPs activating extraintestinal pathogenic *Escherichia coli* (ExPEC) histidine kinase PhoQ. Bacteria (FY26, FY26Δ*phoQ*, and FY26C*phoQ*) were grown in M9 media with 10 mM Mg^2+^ at pH 7.5 or pH 5.0 (acidic pH). [**(F)**, a] The transcription levels of *hlyF* and *Mig-14p* for ExPEC strain FY26 were increased with the inducing signal for mildly acidic pH. Bacteria were grown in M9 media with adding CAMPs or non- CAMPs (10 mM Mg^2+^ at pH 7.5). The transcription levels of *hlyF* and *Mig-14p* with the inducing signal for CAMPs shown in [**(F)**, b]. [**(F)**, c] The signals for acidic pH and CAMPs could additively activate ExPEC PhoQ and upregulate the transcription of *hlyF* operon. Data acquired from at least four independent experiments performed in triplicate, and the mean values ± SEs were shown. The statistically significant differences were determined using Unpaired Student’s *t*-test analysis (***P < 0.01).

The host-induced transcription of *hlyF, Mig-14p*, and *OmpTp* suggested that unknown transcriptional regulators might be involved in controlling these genes expression during ExPEC infection (Figure 1B). To identify this possibility, we analyzed the transcription promoter regions of *hlyF*. As shown in Figure 2A, the putative transcription initiation site of the *hlyF* operon is an A at nt −22 upstream of the start codon, and the −10 and −35 consensus boxes with reasonable spacing were indicated. Inspection of the promoter region of *hlyF* revealed a PhoP-binding box with the sequence 5′-TGTTTA ATAAT TGTTTA-3′ between nt −69 and −53 upstream of the *hlyF* start codon, which matched to the PhoP consensus binding motif “TGTTTA(N5)-TGTTTA” in *E. coli* (Figure 2A). Our results showed that a putative PhoP-binding site was located in the promoter region of *hlyF* and *Mig-14p* operon.

The *phoP* mutant and complemented strains were constructed to determine its effects on the transcription level of *hlyF* operon (Table S1 in Supplementary Material). As shown in Figure 2B, the *phoP* deletion led to a substantial decrease (about 54.6- and 45.9-fold) of *hlyF* and *Mig-14p* transcription (*P* < 0.01) respectively, and there was no obvious effect on *ompT* and *OmpTp* transcription. Moreover, the overexpression of PhoP in FY26C*phoP* compensated the negative effect of *phoP* deletion on *hlyF* and *Mig-14p* transcription (Figure 2B). The results suggested that regulator PhoP was a transcriptional activator of *hlyF*/*Mig-14p* transcription. To further identify the positive effect of PhoP on the expression of HlyF and Mig-14p, the *hlyF*:*lacZ-zeo* fusion transcriptional reporter in ColV plasmid was constructed in the mutant FY26Δ*lacI-Z*. As expected, the β-galactosidase activities showed that PhoP was a positive regulator of LacZ expression since an obvious decrease in Miller Units of *phoP* mutant (about 15.2-fold) (*P* < 0.01) (Figure 2C). We also assessed the effect of *phoP* deletion on HlyF and Mig-14p at the protein level. Western blot result showed that lack of PhoP led to the decreased expression of HlyF and Mig-14p in mutant FY26Δ*phoP* when compared with the wild-type FY26 (Figure 2D).

The EMSA was performed to identify the binding of PhoP to the promoter of *hlyF* and *Mig-14p* operon. The promoter of OmpTp acted as the negative control. The *phoP* gene was cloned into plasmid pET-28a (28, 46), and the fusion proteins PhoP were overexpressed and purified (Figure S2 in Supplementary Material). As shown in Figure 2Ea, the purified non-phosphorylated PhoP could bind and shift the promoter P*_hlyF_* DNA, but no binding was observed in the control OmpTp*_p_* fragment. In addition, mutated P*_hlyF_* DNA with nucleotide deletion at promoter P*_hlyF_* position nt −68 to −63 prevented PhoP binding (Figure 2Eb). These results provided additional support to the hypothesis that the region −75/−59 was a PhoP-binding box. The results demonstrated that PhoP could directly bind to the P*_hlyF_* promoter. Moreover, phosphorylation of PhoP enhanced its binding affinity for P*_hlyF_* promoter. EMSA result showed that an obviously increased efficiency for phosphorylated PhoP to bind and shift the P*_hlyF_* fragment compared with that of non-phosphorylated PhoP (*P* < 0.01) (Figure 2Ec,d).

Recent studies show PhoP/PhoQ has important roles in *Salmonella* survival in macrophages. The histidine kinase PhoQ activity of *Salmonella* can be induced by the antimicrobial factors in macrophage phagosome, such as acidic pH and AMPs, which can destroy and kill the pathogenic bacteria (33, 47). The activated PhoQ enhances the phosphorylation level of PhoP before PhoP upregulates the *Salmonella* virulence factors to resist the phagocytes clearance (32, 47). *Salmonella* PhoQ can be activated *in vitro* by exposure at pH 5.0 or with CAMPs at sub-inhibitory concentrations in growth medium (containing 10 mM MgCl_2_) (32, 33, 47). We next determined whether ExPEC PhoQ could be activated *in vitro* at acidic pH or in the presence of CAMPs. Figure 2F showed that the transcription levels of *hlyF* and *Mig-14p* in wild-type FY26 were increased at mildly acidic pH or after CAMPs treatment. The pH and CAMPs dependent activation was observed at 10 mM MgCl_2_. The transcriptions of *hlyF* and *Mig-14p* were increased 11.2- and 9.2-fold when the pH was switched from 7.5 to 5.0, respectively (*P* < 0.01) (Figure 2Fa). The transcription of *hlyF* and *Mig-14p* increased 8.7- and 7.2-fold by adding CAMPs in growth medium, respectively (*P* < 0.01) (Figure 2Fb). Activation by acidic pH and CAMPs was not observed in the *phoQ* mutant, and enhanced transcription of *hlyF* and *Mig-14p* was also observed in the complemented PhoQ strian (Figure 2F). Previous research confirms that the PhoQ activity was suppressed by the high level of divalent cation (10 mM MgCl_2_) (33, 47). Our results showed that the activated PhoQ by acidic pH and CAMPs in ExPEC strain was independent on the concentration of divalent cation. Furthermore, our results showed that acidic pH and CAMPs could additively activate ExPEC PhoQ and upregulate the transcription of *hlyF* operon (Figure 2Fc). The PhoQ activation at acidic pH was greater with the simultaneous presence of CAMPs, and transcriptions of the *hlyF* and *Mig-14p* further enhanced about 6.5- and 5.6-fold on the basis of acidic condition (pH 5.0), respectively (*P* < 0.01) (Figure 2Fc).

### Functional Analysis of Mig-14p and OmpTp Conferring ExPEC Resistance to the Cationic Antimicrobial Peptides (AMPs)

Due to the selective stress of therapeutic CAMPs, many Gram-negative bacteria have evolved specific mechanisms to resist the bactericidal effects of CAMPs. Moreover, recent reports confirm the CAMP-resistance phenomenon is associated with cross-resistance toward CAMP effectors of host innate immune system and results in persistent infection. AMP/defensin susceptibility tests were conducted to determine whether HlyF, Mig-14p, and OmpTp could resist AMPs bactericidal effect. MICs results revealed that strain FY26 exhibited higher resistance to CAMPs LL-37 and HBD2 than that of RS218, a prototypic NMEC strain without ColV plasmids (39) (Table S3 in Supplementary Material). Deletion of Mig-14p and OmpTp sensitized FY26 to the LL-37 and HBD2 with decreased MICs for these mutants (Table S3 in Supplementary Material). The loss of PhoP reduced the MICs to LL-37 and HBD2 by 16- and 8-fold, respectively. However, the MICs results showed that deletion of HlyF had no effect on FY26 resistance to CAMPs (Table S3 in Supplementary Material). Moreover, the corresponding complemented strains of Mig-14p or OmpTp enhanced the resistance to LL-37 and HBD2 (Table S3 in Supplementary Material). Subsequently, the MICs result showed that Mig-14p expression in the complemented RS218CHlyF/Mig-14p exhibited fourfold and eightfold increased susceptibilities to LL-37 and HBD2, compared with that of wild-type RS218 (Table S3 in Supplementary Material).

**Figure 3:**
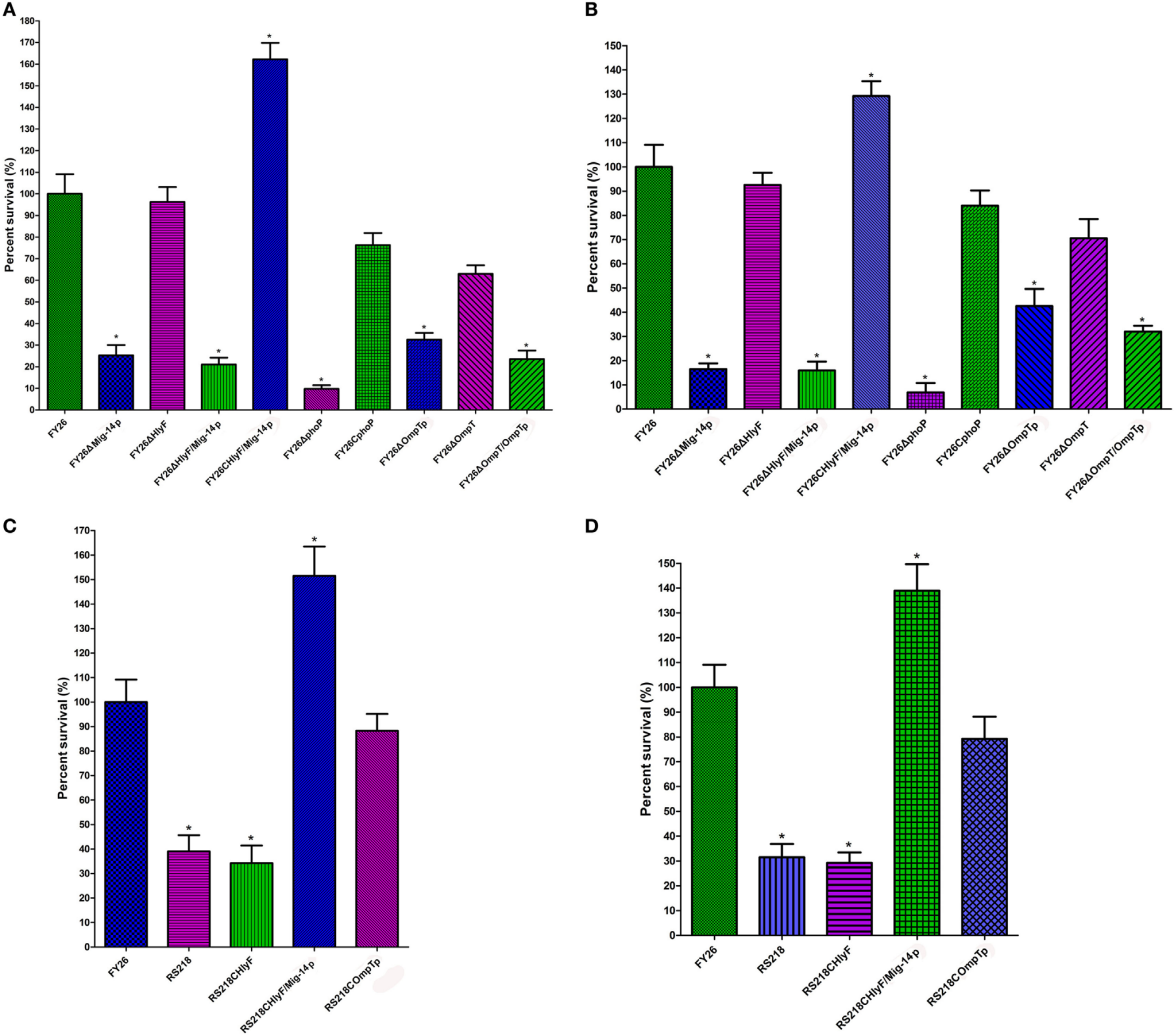
The Mig-14p and OmpTp conferring extraintestinal pathogenic *Escherichia coli* (ExPEC) resistance to CAMPs. CAMPs killing assays were conducted to determine the roles of Mig-14p and OmpTp in ExPEC resistance to CAMPs. **(A)** The sensitivity of wild-type FY26, the Mig-14p mutant, the OmpTp mutant, and complemented strains to LL-37. **(B)** The sensitivity of wild-type FY26, the Mig-14p mutant, the OmpTp mutant, and complemented strains to HBD2. **(C)** CAMPs killing assays were conducted to determine that overexpression of Mig-14p and OmpTp in RS218 increased its resistance to LL-37. **(D)** Overexpression of Mig-14p and OmpTp in RS218 increased its resistance to HBD2. Data are expressed as the percent difference in survival following a 2 h exposure to LL-37. Survival in each assay was normalized to the value of the wild-type FY26. Data acquired from at least four independent experiments, and the mean values ± SEs were shown. The statistically significant differences were determined using one-way ANOVA analysis (*P < 0.01).

Cationic antimicrobial peptides killing assays were further conducted to identify whether Mig-14p and OmpTp could confer ExPEC resistance to CAMPs. As shown in Figure 3A, FY26ΔMig-14p mutant showed increased susceptibility to both LL-37 (25.3%) and HBD2 (16.5%) (*P* < 0.01) compared with the wild-type FY26. FY26ΔOmpTp mutant showed increased susceptibility to both LL-37 (about 32.5% survival) and HBD2 (42.5% survival), respectively (*P* < 0.01). Interestingly, FY26ΔPhoP mutant exhibited higher susceptibility to LL-37 and HBD2 (9.8 and 6.9% survival, respectively, relative to FY26) (*P* < 0.01) (Figure 3B), suggesting that PhoP/PhoQ could mediate ExPEC resistance to CAMPs through more than one regulatory pathways, for example, PhoQ/PhoP can regulate *E. coli* to activate lipopolysaccharide (LPS) modification in increased bacterial resistance to CAMPs (48). By contrast, FY26ΔHlyF showed no increased susceptibility to CAMPs when compared with FY26, which suggested that HlyF was not associated with ExPEC CAMPs resistance (Figure 3A). Furthermore, overexpression of the Mig-14p and OmpTp in FY26 mutants significantly increased the survival when exposed to LL-37 and HBD2. The survival level of the OmpTp complemented strain restored to that of wild-type FY26, and the survival of the complemented Mig-14p was higher than that of FY26 (*P* < 0.01) (Figure 3B). Similarly, the overexpression of Mig-14p and OmpTp in RS218 conferred their resistance to CAMPs (Figure 3C). The survival level of the complemented OmpTp strain RS218COmpTp was higher than that of wild-type RS218, and RS218 only possessed about 44.2% survival to LL-37 and 39.7% survival to HBD2 compared with that of the overexpressed strain RS218COmpTp. The survival of RS218CHlyF/Mig-14p was enhanced about 3.9-fold to LL-37 and 4.4%-fold to HBD2, respectively, relative to that of RS218 (*P* < 0.01) (Figure 3D). Thus, our data indicated that Mig-14p and OmpTp in ColV plasmids had important roles to confer ExPEC resistance to CAMPs, and the resistance effect of Mig-14p was greater than that for OmpTp based on CAMPs susceptibility and killing tests.

### PhoP/PhoQ-HlyF Pathway Was Essential for ExPEC Intracellular Survival in Macrophages

**Figure 4:**
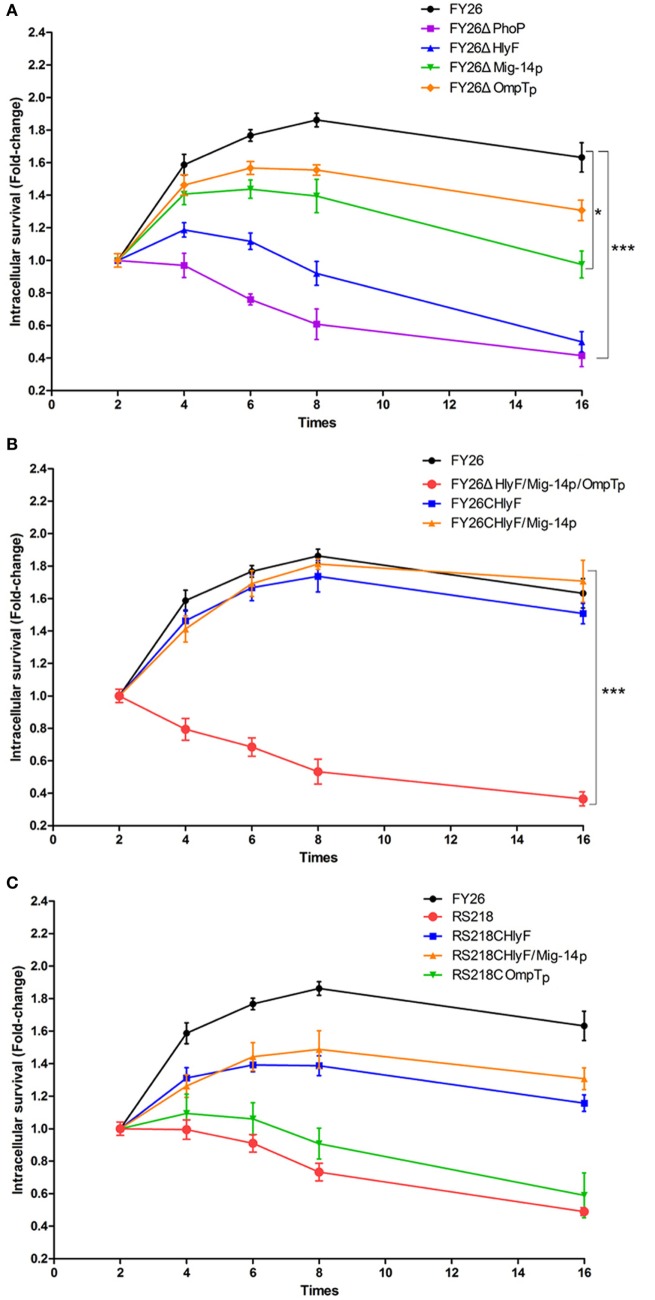
PhoP/PhoQ-HlyF pathway contributed to ExPEC intracellular survival in macrophages. **(A)** To investigate the contribution of HlyF, Mig-14p, OmpTp, and PhoP on FY26 survival within macrophages. The intracellular survival of wild-type FY26 and four mutants were determined, as fold change in intracellular bacterial number at post-infection time points (4, 6, 8, and 16 hpi) compared with the initial number of intracellular bacteria for 2 hpi. **(B)** To determine the survival rates of FY26 and its complemented strains for HlyF, Mig-14p, and OmpTp. **(C)** To determine the survival rates of ColV plasmidless RS218 and its complemented strains for HlyF, Mig-14p, and OmpTp. Data acquired from at least four independent experiments performed in triplicate, and the mean values ± SEs were shown. The statistically significant differences were determined using two-way ANOVA analysis (*P < 0.05; ***P < 0.01).

Previous studies have shown that ExPEC can survive and replicate in macrophages (15, 16, 49). The acidic pH and CAMPs acted as the antimicrobial factors in macrophage phagosome to kill the pathogenic bacteria (31, 32). Our results had confirmed that the two signals (acidic pH and CAMPs) could directly activate ExPEC PhoP/PhoQ, and PhoP could directly regulate the expression of HlyF and Mig-14p. Therefore, HlyF operon presented host-induced transcription during ExPEC infection in macrophages. Moreover, Mig-14p and OmpTp could confer ExPEC resistance to CAMPs. To further investigate the contribution of HlyF, Mig-14p, OmpTp, and PhoP/PhoQ on ExPEC interaction with macrophages, we measured intracellular survival of wild-type FY26, the mutants, and complemented strains. As expected, the intracellular survival of *Mig-14p* and *OmpTp* mutants were impaired in HD11 macrophages compared with wild-type FY26 (*P* < 0.05) (Figure 4A). Meanwhile, the intracellular survival of the mutants lacking the HlyF or PhoP was also significantly impaired when compared with FY26 (*P* < 0.01) (Figure 4A). The survival rates of the *hlyF* mutant at 4 h post-infection (hpi) and 8 hpi were significantly decreased about 31.9 and 24.9% when compared with that of FY26 (*P* < 0.01) (Figure 4A). The survival rates of *phoP* mutant at 4 and 8 hpi were just only 31.9 and 24.9% compared with that of FY26, respectively (*P* < 0.01) (Figure 4A). As shown in Figure 4B, the survival rates of the complemented HlyF and Mig-14p strains were restored to that of wild-type FY26 (*P* > 0.05). Moreover, we determined the survival rates of ColV plasmidless RS218 and its complemented HlyF, Mig-14p, and OmpTp strains. The results showed that survival level of RS218 was obviously lower than that of FY26, and the survival level of the complemented HlyF and pMig strains were significantly higher than that of RS218 (*P* < 0.01) (Figure 4C). But the complement of OmpTp in RS218 had slightly effect on the survival level, compared with RS218 (*P* < 0.05) (Figure 4C). These results clarified that the activity of TCS PhoP/PhoQ was required for ExPEC survival in macrophages. HlyF and Mig-14p acted as intracellular survival factors to promote ExPEC resistance to killing by macrophages.

### PhoP/PhoQ-HlyF Pathway Was Required for ExPEC to Prevent Phagolysosomal Fusion/Acidification and Damage the Phagolysosomal Membranes

**Figure 5:**
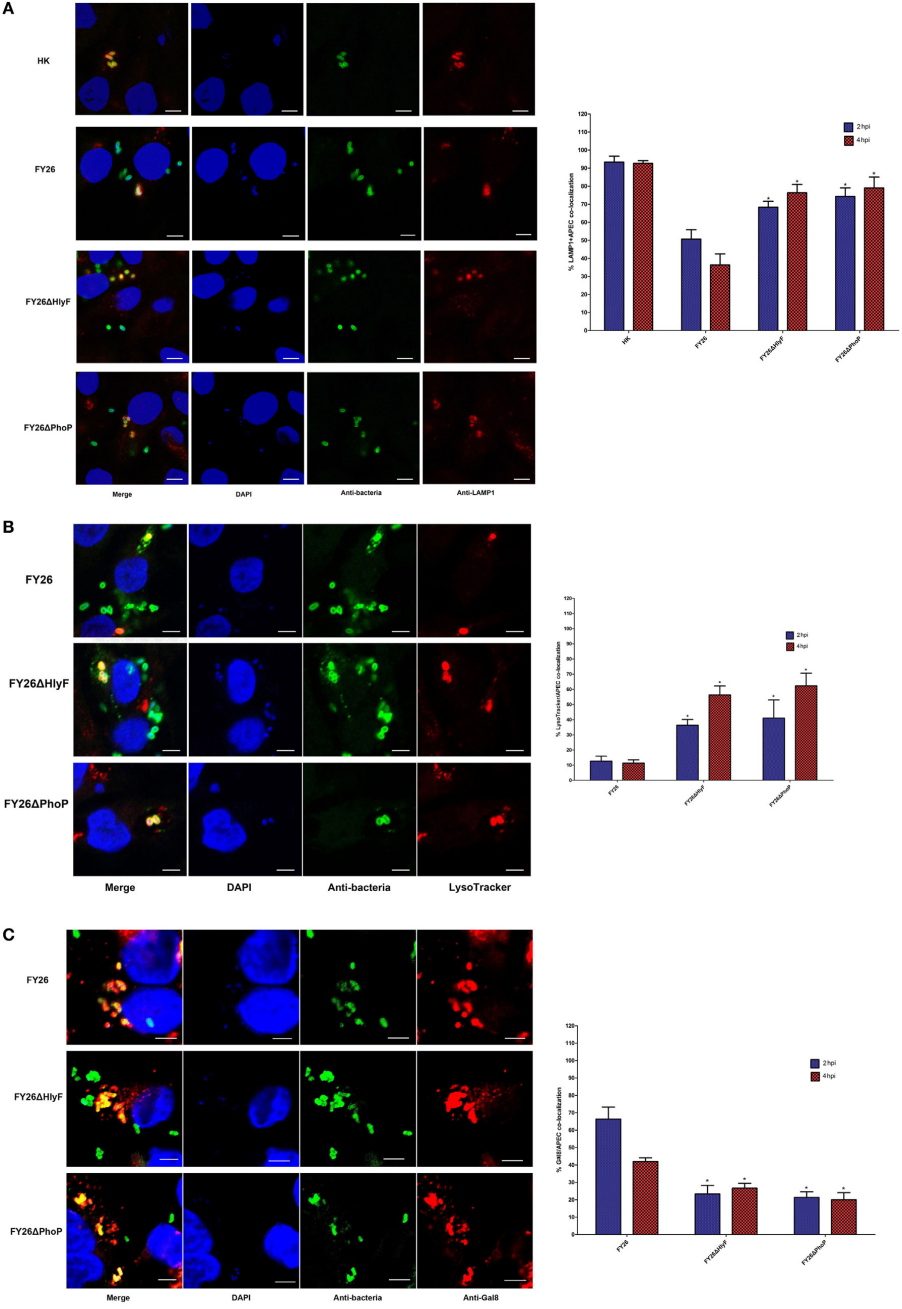
PhoP/PhoQ-HlyF pathway mediated extraintestinal pathogenic *Escherichia coli* (ExPEC) to prevent phagolysosomal fusion/acidification and damage the phagolysosomal membranes. **(A)** To determine the colocalization rates of LAMP1 with live or heat-killing (HK) wild-type FY26, FY26ΔHlyF, and FY26ΔPhoP at a multiplicity of infection (MOI) of 5. Bacteria were labeled with an anti-ExPEC antibody (Alexa 488, green) and anti-LAMP1 antibody (Alexa 568, red), and DNA was dyed with DAPI (blue). Representative confocal microscopy images [the left side of **(A)**] for 2 hpi were shown. Scale bar = 10 µm. Quantification of the colocalization rates of ExPEC strains with LAMP1 at 2 and 4 hpi was shown in right side of **(A)**. **(B)** To determine the colocalization rates of acidotropic probe LysoTracker Red with wild-type FY26, FY26ΔHlyF, and FY26ΔPhoP at an MOI of 5. Bacteria were labeled with an anti-ExPEC antibody (Alexa 488, green) and LysoTracker Red (red), and DNA was dyed with DAPI (blue). Representative confocal microscopy images [the left side of **(B)**] for 2 hpi were shown. Scale bar = 5 µm. Quantification of the colocalization rates of ExPEC strains with LysoTracker Red at 2 and 4 hpi was shown in right side of **(B)**. **(C)** To determine the colocalization rates of galectin 8 (Gal8) with wild-type FY26, FY26ΔHlyF, and FY26ΔPhoP at an MOI of 5. Bacteria were labeled with an anti-ExPEC antibody (Alexa 488, green) and anti-Gal8 antibody (Alexa 568, red). DNA was dyed with DAPI (blue). Representative confocal microscopy images [the left side of **(C)**] for 2 hpi were shown. Scale bar = 5 µm. Quantification of the colocalization rates of ExPEC strains with Gal8 at 2 and 4 hpi was shown in right side of **(C)**. Data for quantification of the colocalization rates represented the results of more than 100 infected HD11 cells in at least three independent tests, and the mean values ± SEs were shown. The statistically significant differences were determined using one-way ANOVA analysis (*P < 0.01).

After phagocytic uptake of bacteria by lysosomes, the essential micro-bicidal mechanism depends on fusion of microbe-containing lysosomes to the phagosomes and the formation of the microbicidal phagolysosomes (50–52). To infect successfully after breaking through host barriers, pathogens have developed various strategies to resist phagocytic killing in macrophages (36, 53–55). However, the intracellular survival mechanism associated with ExPEC pathogenesis is not well-understood. To better understand how ExPEC survive in macrophages, we investigated roles of PhoP/PhoQ-HlyF pathway in promoting ExPEC resistance to macrophages killing. The immunofluorescence labeling was performed to identify the intracellular localization of ExPEC in macrophages after phagocytosis. To determine whether ExPEC-containing vesicle formation depended on the classical process of phagolysosomes maturation, the colocalization with LAMP1 and bacteria within macrophages was detected. The heat-killed (HK) bacteria were used as a positive control, and over 90% of heat-killed (HK) bacteria were colocalized with LAMP1 at 2 and 4 hpi, respectively (*P* < 0.01) (Figure 5A). The colocalization rates of LAMP1 with wild-type bacteria were approximately 50.7 and 36.3% under the infection of FY26 in HD11 macrophages at 2 and 4 hpi, respectively (*P* < 0.01) (Figure 5A). In HlyF-deficient mutant infected cells, about 68.3 and 76.3% of labeled ExPEC bacteria were associated with LAMP1 at 2 and 4 hpi after uptake in HD11, respectively. Compared with wild-type FY26, the association with LAMP1 was significant enhanced after *hlyF* deletion (*P* < 0.01) (Figure 5A). As anticipated, the colocalization between LAMP1 and the *phoP* mutant was significant enhanced about 74.3 and 79.0% at 2 and 4 hpi after exposed to HD11, respectively (*P* < 0.01) (Figure 5A). The enhancement of the colocalization after HlyF and PhoP deletion suggested that PhoP/PhoQ-HlyF pathway might prevent the fusion of ExPEC-containing phagosomes with lysosomes.

In above section, we showed PhoP/PhoQ-HlyF pathway was essential for ExPEC intracellular survival, suggesting that PhoP/PhoQ-HlyF interfered with phagolysosomal killing. The phagolysosomal acidification by a markedly acidic pH (pH 4.5–5) is required for the mature phagolysosomes to achieve effectively bacterial killing and degradation (54, 55). An acidotropic probe dye (LysoTracker Red DND-99) was used to label and track the acidic phagolysosomes. As shown in Figure 5B, the colocalization rates of LysoTracker probe with wild-type bacteria were only 12.7 and 11.3% in FY26-infected HD11 macrophages at 2 and 4 hpi, respectively (*P* < 0.01). In HlyF-deficient mutant infected cells, more than 36.3 and 56.3% of labeled ExPEC bacteria had undergone acidification at 2 and 4 hpi. As anticipated, the colocalization with LysoTracker probe for PhoP mutant was significantly enhanced about 41.0 and 62.3% at 2 and 4 hpi, respectively (*P* < 0.01) (Figure 5B). The association with acidic phagolysosomes was significantly increased due to *hlyF* and *phoP* deletion during the ExPEC infection (*P* < 0.01) (Figure 5B). The finding further identified that PhoP/PhoQ-HlyF pathway could prevents phagolysosomal acidification.

The lower colocalization of wild-type FY26 with LAMP1-positive phagolysosomes during its infection suggested that wild-type FY26 could escape from phagolysosomes and enter the cytosol. To further evaluate whether PhoP/PhoQ-HlyF pathway was involved in membrane damage in the ExPEC-containing vesicle, the bacteria within macrophages was detected for their colocalization with Gal8, a cytosolic lectin used as the label for vesicle membranes damage. The vesicle membrane contains lots of galactosides in its interior surface, and Gal8 can directly bind galactosides (54). When the vesicle membrane is damaged, these galactosides are leaked into the cytosol, thereby promoting Gal8 binding to the bacterial-containing damaged vesicle (56). As shown in Figure 5C, the colocalization of Gal8 with wild-type bacteria was more than 66.3 and 42.0% in FY26-infected HD11 macrophages at 2 and 4 hpi, respectively (*P* < 0.01). In HlyF mutant infected cells at 2 and 4 hpi, only 23.3 and 26.7% of labeled ExPEC bacteria colocalized with Gal8, respectively. As expected, the colocalization rates with Gal8 for PhoP mutant was merely about 21.2 and 19.8% at 2 and 4 h (*P* < 0.01) (Figure 5C). The association with Gal8 was significantly decreased after *hlyF* and *phoP* deletion during ExPEC infecting macrophages (*P* < 0.01) (Figure 5B). The finding supported that PhoP/PhoQ-HlyF was involved in bacterial-containing vesicle membrane damage, thereby preventing phagolysosomal fusion and acidification and promoting ExPEC to escape into the cytosol.

### PhoP/PhoQ-HlyF Pathway Promoted the Formation of ExPEC-Containing Autophagosome During Survival in Macrophages

**Figure 6:**
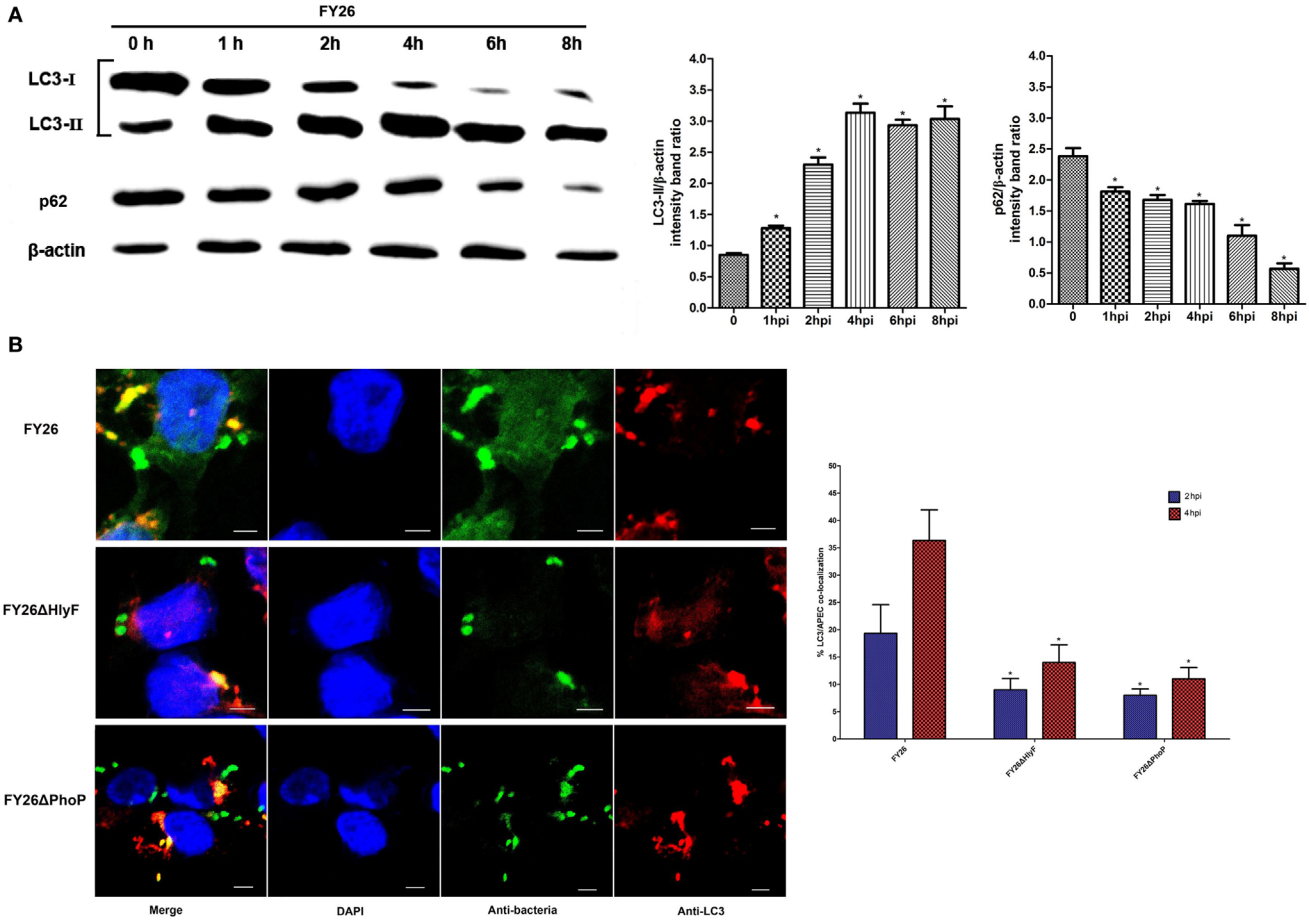
PhoP/PhoQ-HlyF pathway promoted the formation of extraintestinal pathogenic *Escherichia coli* (ExPEC)-containing autophagosome during survival in macrophages. **(A)** Western blots of LC3 and p62 in FY26-infected HD11 cells. HD11 cells were infected with FY26 at multiplicity of infection (MOI) of 50. At 1, 2, 4, 6, or 8 h post-infection (hpi), the cells were lysed, and SDS-PAGE was performed. Western blot using antibodies against LC3, p62, and β-actin protein as indicated. Densitometry analysis was performed to relative quantification. The data for the ratio of LC3-II and p62 to β-actin were acquired from three independent experiments, and the mean values ± SEs were shown. The statistically significant differences were determined using one-way ANOVA analysis (*P < 0.01). **(B)** To determine the colocalization rates of LC3 with wild-type FY26, FY26ΔHlyF, and FY26ΔPhoP at an MOI of 5. Bacteria were labeled with an anti-ExPEC antibody (Alexa 488, green) and anti-LC3 antibodies (Alexa 568, red). DNA was dyed with DAPI (blue). Representative confocal microscopy images (the left side of Figure 6B) for 2 hpi were shown. Scale bar = 5 µm. Quantification of the colocalization rates of ExPEC strains with LC3 at 2 and 4 hpi was shown in right side of Figure 6B. Data for quantification of the colocalization rates represented the results of more than 100 infected HD11 cells in each of least three independent tests, and the mean values ± SEs were shown. The statistically significant differences were determined using one-way ANOVA analysis (*P < 0.01).

Many invasive bacteria can survive and proliferate in professional phagocytes upon its infection. These pathogens sometimes escape from bacteria-containing vesicles and enter the cytosol. During infection in the cytosol, pathogens induce and activate macroautophagy (microorganism-specific autophagy), which is a critical innate immune response pathway to target and degrade intracellular microorganisms (57–59). The autophagosomes (the typical double membrane compartments) are established during the autophagy, and the autophagy adaptor protein LC3 and p62 act as the typical markers for bacteria-containing autophagosome formation (57, 60). Extensive works confirm that the LC3-I is gradually converted into LC3-II during the activated autophagy in response to bacterial infection, and autophagy-recruited LC3-II colocalizes with bacteria-containing autophagosomes. Simultaneously, the p62 is gradually degraded during autophagosome formation, and decreased p62 expression acted as the critical index for testing autophagic degradation (50, 52). Eventually, LC3-decorated autophagosomes are delivered into the lysosome to form degradative autolysosomes during antibacterial autophagy process (50, 51). To investigate whether ExPEC infection could induce macrophages autophagic response, we analyzed LC3 and p62 of HD11 macrophages after infected with FY26 by Western blotting. The autophagy level was determined by the ratio of LC3-II/β-actin band intensity among different time points of post-infection. As a control of autophagy induction, the western blot for HD11 cells without infection or pretreated with rapamycin was shown in Figure S3 in Supplementary Material. The result showed that expression of LC3-II was gradually increased in infected cells from 1 to 8 hpi, and the ratio of p62/β-actin intensity showed the p62 expression level in HD11 cells was gradually decreased (*P* < 0.05) (Figure 6A). Another key hallmark of the autophagy is Ub, and poly-ubiquitination acts as a tag for antibacterial autophagy (36, 61). The immunofluorescence labeling was performed to further identify the autophagy activity in FY26-infected HD11 macrophages. As shown in Figure S4 in Supplementary Material, the representative confocal microscopy image showed that LC3-labeled bacteria-containing autophagosomes were colocalized with Ub (*P* < 0.05). Together, these findings indicated that autophagy was activated during ExPEC infection.

The above studies showed that PhoP/PhoQ-HlyF was involved in the membrane damage of bacterial-containing vesicle and promoted ExPEC to escape into the cytosol. Due to autophagy in response to ExPEC infection, we further evaluated whether PhoP/PhoQ-HlyF pathway influenced the antibacterial autophagy response in FY26-infected HD11 cells. As shown in Figure 6B, the colocalization rates of LC3 with wild-type bacteria were more than 19.4 and 36.3% in FY26-infected HD11 macrophages at 2 and 4 hpi, respectively (*P* < 0.01), suggesting approximately 19.4 and 36.3% (at 2 and 4 hpi, respectively) of intracellular bacteria could be targeted for antibacterial autophagy. In HlyF-deficient mutant infected cells, only 9.0 and 14.2% of labeled ExPEC bacteria were colocalized with LC3 at 2 and 4 hpi. As expected, the colocalization rates with LC3 in PhoP-deficient mutant were merely about 8.1 and 11.0% at 2 and 4 hpi, respectively (*P* < 0.01) (Figure 6B). The association with LC3 was significantly decreased due to *hlyF* and *phoP* deletion during ExPEC infection (*P* < 0.01) (Figure 6B). The finding suggested that PhoP/PhoQ-HlyF pathway might promote the formation of ExPEC-containing autophagosome in macrophages. However, autophagy, known as an antibacterial defense, might be insufficient to control ExPEC infection, as shown by the presence of ExPEC intracellular replication in macrophages.

### PhoP/PhoQ-HlyF Signaling Pathway Upregulating the Production of ExPEC OMVs

**Figure 7:**
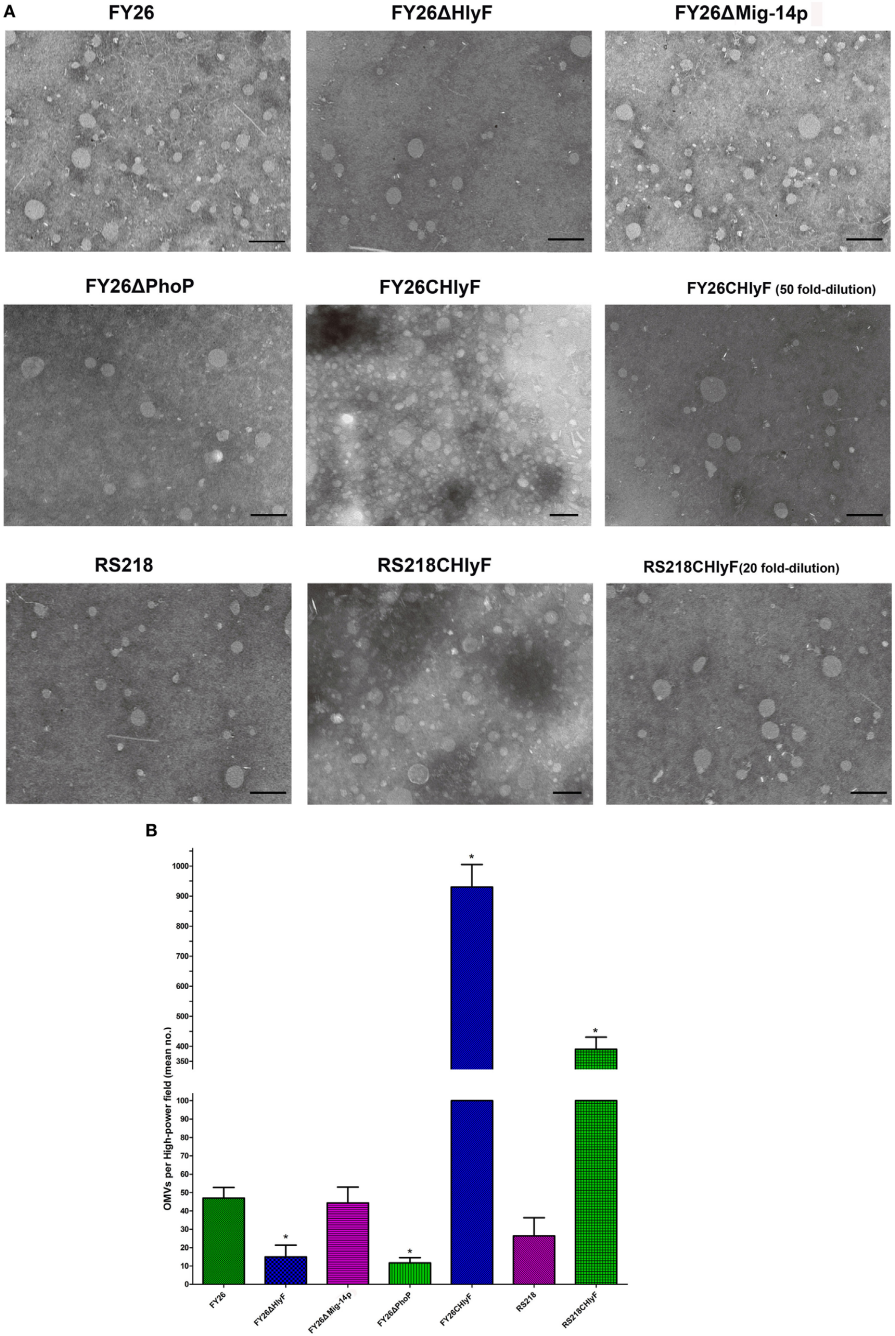
PhoP/PhoQ-HlyF pathway upregulating the production of extraintestinal pathogenic *Escherichia coli* (ExPEC) outer membrane vesicles (OMVs). Strains FY26, FY26ΔHlyF, FY26ΔPhoP, FY26ΔMig-14p, FY26CHlyF, RS218, and RS218CHlyF were cultured in LB at 37°C for 16 h. The OMVs in each strain filtrated supernatant (500 ml) were precipitated by high-speed centrifugation and resuspended with 50 µl Tris–HCl buffer. **(A)** The isolated OMVs of each strain were visualized by transmission electron microscopy (TEM) under 100,000× original magnification. For Bar: 200 nm. The OMVs for FY26CHlyF (50-fold dilution) and RS218CHlyF (20-fold dilution) were also detected by TEM under the same view conditions. **(B)** Quantification of the production of OMVs for ExPEC strains. More than 10 images (high-power field) for each strain were used to quantify the OMVs. Data acquired from at least four independent experiments performed in triplicate, and the mean values ± SEs were shown. The statistically significant differences were determined using one-way ANOVA analysis (*P < 0.01).

Outer membrane vesicles play critical roles in pathogenesis and intercellular interactions of Gram-negative pathogens (62, 63). Many bacterial species release OMVs, including *Salmonella* sp., *E. coli, Legionella pneumophila*, and *Pseudomonas aeruginosa*. OMVs (10–300 nm small particles) are secreted from the bacterial outer membrane and mainly contain lipids, LPS, OM proteins, and cell wall components (23, 62, 63). Moreover, OMVs also contains periplasmic, inner membrane, cytoplasmic or secreted virulence factors and toxins, which can be transmitted to host cells and thereby involved in modulation of host immune response, adherence, antibiotic resistance, and others (63). One study shows that *L. pneumophila*-derived OMVs are essential for its survival and replication in macrophages (44). Several mechanisms for OMVs production are identified, for example, LPS remodeling promotes OMVs production in *Salmonella* (36), but the general secretion mechanism of OMVs is lacking. Murase et al. identifies that the virulence factor HlyF is essential for ExPEC OMVs formation. Therefore, we next identified whether PhoP/PhoQ regulated the production of ExPEC OMVs. The strain FY26 and its variants for *phoP, hlyF*, and *Mig-14p* deletion mutants were used to determine the production of OMVs, as well as the RS218 and complemented RS218CHlyF. The OMVs of ExPEC were detected by TEM under the same conditions. The size range of OMVs for FY26 and RS218 was similar to these mutants, ranging from 20 to 150 nm, which was consistent with the report of Murase et al. (Figure 7A). Quantification of the OMVs for each strain was conducted, and the amount of OMVs (about 47 under 100,000× original magnification) in wild-type FY26 was higher than that in strain RS218 (about 26 per high-power field) (*P* < 0.01) (Figure 7B). The OMV production levels in the supernatant of FY26ΔHlyF and FY26ΔPhoP were obviously decreased about 31.9 and 24.9%, compared with that of FY26 (*P* < 0.01) (Figure 7B). The deletion of Mig-14p had no effect on the FY26 OMVs production. Moreover, the OMV production in HlyF overexpressing strains (FY26CHlyF and RS218CHlyF) reached to a higher level (mean no. about 930 and 394, respectively) compared with that of FY26 and RS218, respectively (*P* < 0.01) (Figure 7B). Considering that PhoP/PhoQ directly regulated the transcriptional expression of HlyF operon, we concluded that PhoP/PhoQ might trigger OMVs biogenesis and control the production of ExPEC OMVs by regulating the HlyF expression. Bacterial OMVs contain secreted virulence factors to modulation of host immune response (23, 62, 63). Since PhoP/PhoQ-HlyF signaling pathway regulated the production of ExPEC OMVs, OMVs might play critical roles in ExPEC survival and replication in macrophages.

## Discussion

To infect the host, the pathogen predominantly relies on the adhesion, invasion, and survival abilities. More and more evidences suggest that ExPEC is a primary pathogen rather than opportunistic pathogens to infect humans and animals (6–9). To successfully establish the infection after breaking through host barriers, ExPECs must evolve several novel mechanisms to replicate in macrophages. Like the mobile pathogenicity islands, acquisition of large plasmids by pathogen acts as another convenient and effective way to adapt to specific host environment during the infection, and these plasmids can carry multiple adaptability factors, including multiple drug resistance, virulence, and fitness in host niches (64, 65). ColV plasmids undertake significant roles to increase the outbreak rate and lethality of ExPEC infections. Although recent studies show the roles of large plasmids in ExPEC virulence, little is known about whether the ColV plasmids are associated with ExPEC survival and persistence in macrophages.

In this study, molecular characteristics of HlyF, Mig-14 ortholog (Mig-14p), and OmpT variant (OmpTp) encoded by ColV plasmids were identified. HlyF is first discovered as a potential hemolysin protein in APEC, and the recent report by Murase et al. clearly points out that virulence factor HlyF mediates production of ExPEC OMVs (23). We confirmed that HlyF was also located in the cytoplasm of strain FY26 (dominant serotype O2:K1). The sequence alignment showed Mig-14p was a novel Mig-14 ortholog in ExPEC, and it was the second reported Mig-14-like protein, which was similar to protein PA5003 in *P. aeruginosa* required for AMP recognition (38). Unlike the single transcriptional unit of *mig-14* gene in *S. enterica*, the *Mig-14p* and *hlyF* in ExPEC ColV plasmids belong to one operon (25). Furthermore, our immunoblotting and immunofluorescent results showed that Mig-14p also acted as an inner membrane-related protein in ExPEC. OmpTp was another novel identified OmpT variants in pathogenic bacteria and also located in the outer membrane of ExPECs (27, 28).

Currently, AMPs are considered to be one of the most promising antibiotic medicine, due to the severe multidrug resistance to conventional antibiotics. Most AMPs, such as cathelicidin LL-37, belong to cationic AMPs (CAMPs) and are short amphipathic peptides (26, 66). CAMPs can bind to the bacterial membrane surface and permeate or integrate into the cytoplasmic membrane to form the pores and kill the targeted pathogenic microorganisms. When CAMPs enter the bacterial cytoplasm, they can destroy bacterial metabolic components and inhibit the synthesis of critical proteins, nucleic acids, and the cell wall components (26, 67).However, due to the selective stress of thera-peutic CAMPs in recent years, many reports describe that pathogens have evolved defense strategies to resist CAMP-mediated killing. Surface charge modification is an important defense strategy to inhibit the binding of cationic AMPs to bacteria membrane. LPS modification to increase resistance to CAMPs is the major mechanism responsible for surface charge modification in Gram-negative bacteria, such as *E. coli* and *Salmonella*, which regulate the modification of LPS through PhoP/PhoQ sensing and activation (30, 31).

Inactivating and cleaving CAMPs by membrane-related proteases is another mechanism used by bacteria to inhibit CAMPs integration into the bacterial cytoplasm. The previous reports have shown that OmpT proteases are typical outer membrane proteins to degrade host-derived CAMPs, and Mig-14 is an inner membrane-associated protein to facilitate *Salmonella* resistance to CAMPs (25, 27, 28). Like OmpT and Mig-14, our study had identified that ColV plasmid-encoded Mig-14p and OmpTp played important roles in ExPEC resistance to CAMPs, and might inhibit the penetration of bacterial membranes by CAMPs. However, unlike OmpT in enterohemorrhagic and enteropathogenic *E. coli*, chromosome-encoded OmpT in ExPEC strain FY26 had no obvious effect on AMP-resistance, which might be caused by the lower transcription level of OmpT in FY26 (27, 28).

**Figure 8:**
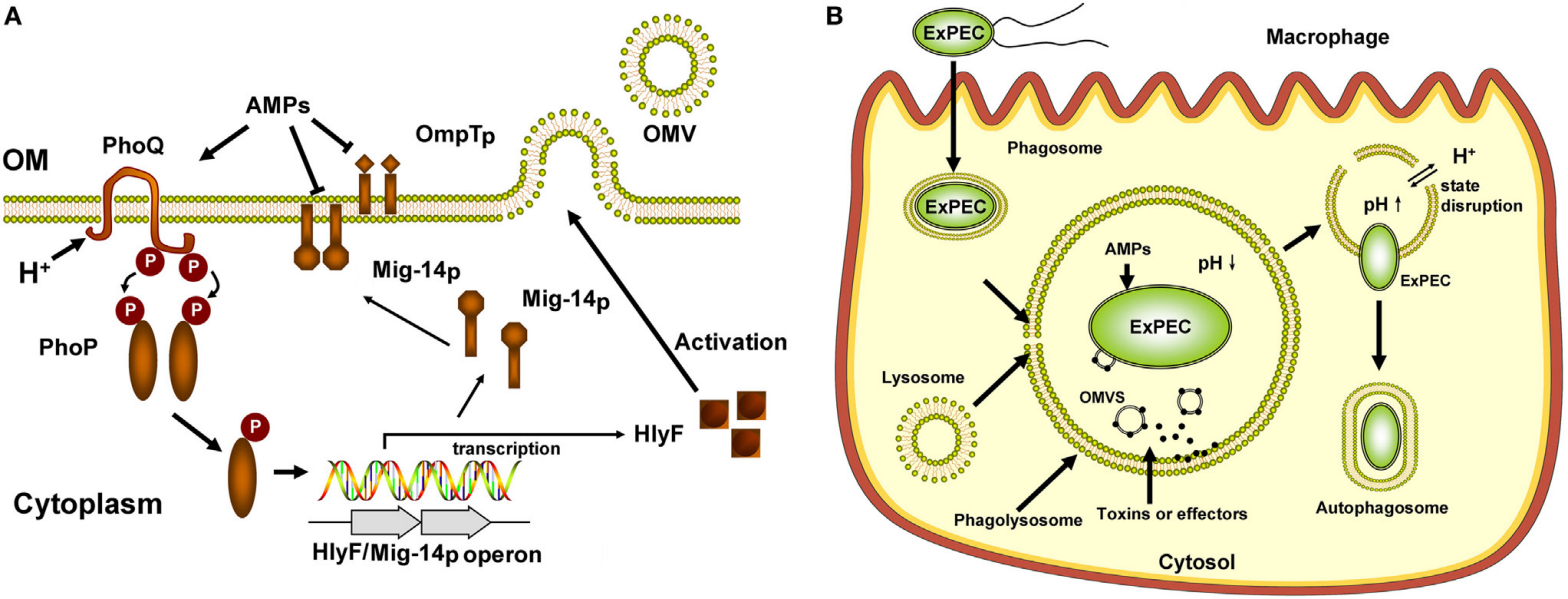
Illustration of PhoP/PhoQ-regulated phenotypes modulating extraintestinal pathogenic *Escherichia coli* (ExPEC) intracellular trafficking in macrophages. **(A)** Summary of PhoP/PhoQ-regulated phenotypes in ExPEC. In response to critical components of bacterial killing within macrophages, PhoQ was activated by CAMPs and acidic pH for ExPEC survival within macrophage phagolysosomes. The activated PhoQ enhances the phosphorylation level of PhoP, and phosphorylated PhoP enhanced its binding affinity for P*_hlyF_* promoter to upregulate the transcription of HlyF and Mig-14p (a novel PhoP regulon). Mig-14p, located in inner membrane, conferred ExPEC resistance to CAMPs. Mig-14p and OmpTp inhibited the penetration of bacterial membrane by CAMPs. PhoP/PhoQ triggered outer membrane vesicles (OMVs) biogenesis and controls the production of ExPEC OMVs by upregulating the HlyF expression. **(B)** Model of ExPEC intracellular trafficking in macrophages. ExPEC was internalized by phagocytosis. The ExPEC-containing phagosomes fused with lysosomes phagolysosomes. PhoP/PhoQ-HlyF signaling pathway regulated the production of ExPEC OMVs, and OMVs might play critical roles in ExPEC to prevent bacterial-containing phagolysosomal fusion and phagolysosomal acidification. ExPEC OMVs contain some unknown toxins and effectors, which might be associated with ExPEC-containing phagolysosomal membrane disruption. PhoP/PhoQ-HlyF pathway promoted the escape of ExPEC from phagolysosomes and entry into macrophage cytosol and facilitated the formation of ExPEC-containing autophagosome. ExPEC might hijack the autophagy to promote its intracellular replication.

Cationic antimicrobial peptides are an integral part of host innate immune system in most multi-cellular organisms. CAMPs are recruited to phagolysosomes and act as a critical component of bacterial killing within macrophages against bacterial infection. Moreover, recent reports confirm the CAMP-resistance phenomenon at the same time is associated with cross-resistance toward host innate immune system and results in persistent infection (68, 69). Mig-14 plays an important role in the survival of *Salmonella* within macrophages. Our study showed that Mig-14p and OmpTp acted as intracellular survival factors to promote ExPEC resistance to killing by macrophages. But the intracellular survival effect for Mig-14p was greater than that of OmpTp. Recent studies demonstrate that CAMPs act as the inducible signal to activate the two-component regulatory system. The typical TCS CsrRS plays critical roles in oropharyngeal colonization and persistence of group A *Streptococcus* (GAS) through upregulation of virulence factors. CsrS can specifically sense the LL-37 signal by directly binding and activating the regulator CsrR (70), and LL-37 can enhance the GAS resistance to killing by host cells (71). Unlike CsrS, PhoP/PhoQ can be activated by the broad repertoire of CAMPs (72). Our study further identified that PhoQ in ExPEC strain could be activated by different CAMPs and further upregulated the expression of Mig-14p (Figure 8A). Since PhoQ is widely distributed in Gram-negative bacteria, it might evolve to sense multiple types of CAMPs.

The phagosome acidification by an acidic pH (4.5–5) is the critical step in phagolysosomes maturation to achieve effective bacteria killing and degradation (54, 55). Within the phagolysosomes, the bacteria are exposed to an acidic environment and high levels of antimicrobial molecules (53). Except for the activation of sensor kinase PhoQ by CAMPs, PhoQ activity is also induced by acidic pH. Unlike the CAMPs and divalent cation (Mg^2+^) that bind the acidic surface of the PhoQ sensor, environmental acidic pH is sensed through the PhoQ cytoplasmic domain (32, 33, 47). The PhoP/PhoQ system is best studied in *Salmonella*. The acidic pH and CAMPs sensing by PhoQ are dispensable for *Salmonella* virulence to replication within macrophage (30). In this study, our results unraveled that ExPEC PhoQ could be activated by low pH *in vitro*, and transcription levels of *hlyF* and *Mig-14p* of ExPEC strain FY26 were increased under mildly acidic pH or in the presence of CAMPs. Our results further showed that the activation of PhoQ was independent of divalent cation concentration, and acidic pH and CAMPs could additively activate ExPEC PhoQ to upregulate the transcription of the *hlyF* operon (Figure 8A).

PhoP/PhoQ system promotes ExPEC pathogenicity when infects the urinary tract and avian, and regulator PhoP mediates *E. coli* virulence and membrane modification. PhoP differentially regulates the transcription levels of hundreds of ExPEC genes involved in resistance to AMPs, invasion/adhesion, repression of motility, LPS modification, acidic pH adaptability, and oxygen-independent changes (31, 73). Our research first identified that PhoP/PhoQ was essential for ExPEC intracellular survival in macrophages (Figure 8B). Since CAMPs, acidic pH, and the oxidative burst are required for macrophage phagolysosomes to perform bacterial killing, the functions of PhoP described by Alteri et al. might promote ExPEC intracellular survival. In this study, the roles of ColV plasmid–encoded virulence factors HlyF, Mig-14p, and OmpTp had been established to be required for ExPEC intracellular survival. We identified the critical correlation (a novel PhoP/PhoQ regulatory pathway) between PhoP/PhoQ system and its regulon (HlyF and Mig-14p), which played the important roles in ExPEC replication within macrophages. The results provided new insights into the roles of CloV plasmids in ExPEC virulence.

PhoP/PhoQ-HlyF pathway promoted the escape of ExPEC from phagolysosomes and entry into macrophage cytosol, our studies showed that ExPEC activated macroautophagy (57, 60), since immunofluorescence labeling study demonstrated LC3-labeled ExPEC-containing autophagosome colocalized with Ub, a tag for antibacterial autophagy to decorate cytosolic bacteria (36, 61). The colocalization of LC3 with wild-type ExPEC and the mutants suggested that PhoP/PhoQ-HlyF pathway might promote the formation of ExPEC-containing autophagosome during its survival in macrophages. The macroautophagy is a critical innate immune response pathway to target and degrade intracellular microorganisms (57–59). However, the autophagy activity might be insufficient to control ExPEC infection in macrophages, suggesting that ExPEC might hijack the autophagy to promote its intracellular replication. ExPEC can actively exploit autophagy to facilitate its urinary tract infection, and a key autophagy protein Atg16L1 deficiency confers protection host against ExPEC infection (74). Some bacteria even exploit the autophagy machinery for intracellular infection by inducing autophagy and sometimes blocking formation of degradative autolysosomes to exploit autophagosomes as replicative niches, such as GAS, *Brucella abortus*, and *Yersinia* (58, 75). Due to an incompletely understanding about ExPEC-autophagy interplay, whether ExPEC virulence factors facilitate the ExPEC replication in macrophages by hijacking the autophagy machinery needs further investigate.

Murase et al. clearly points out HlyF is the virulence factor to mediate the production of ExPEC OMVs (23). Our studies revealed that PhoP/PhoQ-HlyF signaling pathway upregulates the production level of ExPEC OMVs. Our data also revealed that TCSs directly controlled OMVs production. HlyF is a putative dehydrogenase/reductase (SDR) located in *E. coli* cytoplasmic, and the SDR catalytic domain contributes to ExPEC OMV formation (23). A new study shows that a lipid A deacylase PagL is involved in *Salmonella* OMVs formation. Lipid A constitutes the hydrophobic anchor of LPS in the bacterial membrane outer leaflet, and LPS constitutes the basic frame structure of OMVs. The lipid A deacylation by PagL leads to LPS remodeling to trigger *Salmonella* OMV formation (76). However, the mechanism for HlyF activity to trigger the formation of ExPEC OMVs was yet to be elucidated. Due to SDR enzymes involved in the synthetic metabolism of lipid, amino acid, and carbohydrate, we speculated that HlyF might participate in the biosynthesis or modification of LPS components. OMVs act as the important delivery vectors of bacteria, and OMV-associated components, such as toxins and effectors, are involved in modulation of host im-mune response, adherence, antibiotic resistance, and others (63). *L. pneumophila*-derived OMVs are more extensively studied, and a proteomic analysis shows that *L. pneumophila* OMVs contain about 70 proteins. Several OMVs-secreted effector proteins are involved in *L. pneumophila* survival and replication within macrophages by inhibition of phagosome-lysosome fusion (45). Moreover, *L. pneumophila* OMVs contain lots of regulatory small RNAs that could impact the immune response during host-pathogen interaction (44). The proteomic analysis of OMV components of enterohemorrhagic *E. coli* (EHEC O157:H7) shows a cocktail of toxins and effectors, containing Stx2a, CdtV, hemolysin, and proteases, which cause host cell injury and apoptosis *via* OMVs intracellular delivery (77).

We found that ExPEC PhoP/PhoQ-HlyF pathway appeared to be an effective way to prevent bacterial-containing phagolysosomal fusion and interfere with phagolysosomal acidification. The colocalization of the acidotropic probe LysoTracker showed that highly virulent ExPEC sabotaged phagolysosomes acidification (Figure 8B). Because PhoP/PhoQ-HlyF signaling pathway regulated the production of ExPEC OMVs, OMVs might play critical roles in ExPEC to prevent phagolysosomal fusion and acidification. ExPEC OMVs might contain some unknown toxins and effectors to modulation of host immune response, and proteomic profiling of ExPEC OMVs need to be further identified. The colocalization of Gal8 with wild-type ExPEC and the mutants showed that PhoP/PhoQ-HlyF pathway was associated with the membrane damage of ExPEC-containing phagolysosomes. Like the EHEC O157:H7 causing host cell injury *via* OMVs intracellular delivery, we spectated that the unknown toxins or effectors within ExPEC OMVs might be involved in the sublytic membrane disruption.

## Author Contributions

JD, XZ, and FX conceived the study and designed the experiments, while YS, FT, JR, DL, JW, and MJ performed research and analyzed data. XZ wrote the manuscript. All the authors have read, critically revised, and approved the final manuscript.

## Conflict of Interest Statement

The authors declare that the research was conducted in the absence of any commercial or financial relationships that could be construed as a potential conflict of interest.

## Supplementary Material

Figure S1 Sequence comparison of **(A)** Mig-14p and ortholog (Mig-14) in *Salmonella* and **(B)** alignment of OmpTp and the homolog of *Escherichia coli* chromosome-encoding OmpT.

Figure S2 Purification of MBP::Mig-14p **(A)**, GST::HlyF **(B)**, OmpTp **(C)**, and PhoP **(D)** fusion proteins expressed in *Escherichia coli* BL21(DE3). Proteins from the total bacterial extracts (lanes 1 and 2) and the purified fusion proteins (lane 1) were separated on an SDS-PAGE with Coomassie, respectively. Lane M, protein marker.

Figure S3 Western blots of LC3 and p62 in uninfected HD11 cells. HD11 cells were treated with rapamycin for 4 h as a positive control of autophagy induction, and incubated with fetal bovine serum-free RPMI 1640 media as the negative control. At 0, 2, 4, or 8 h post, the uninfected cells were lysed, and SDS-PAGE was performed. Western blot using antibodies against LC3, p62, and β-actin protein as indicated.

Figure S4 The immunofluorescence labeling was performed to identify the intracellular localization of LC3 and ubiiquitin (Ub) with wild-type FY26 at a multiplicity of infection of 5. Bacteria were labeled with anti-LC3 antibodies (FITC, green), anti-ExPEC antibody (Alexa 647, purple), and anti-Ub antibodies (TRITC, red). DNA was dyed with DAPI (blue). Representative confocal microscopy images for 4 hpi were shown. Scale bar = 5 µm.

Table S1 Bacterial strains and plasmids used in this study.

Table S2 Oligonucleotide sequences used as PCR primers in this study.

Table S3 MICs of antimicrobial peptides (AMPs) (LL-37 and HBD2).
