# Supplementary figures and images for: A Novel PhoP/PhoQ Regulation Pathway Modulates the Survival of Extraintestinal Pathogenic Escherichia coli in Macrophages
Source: Front Immunol. 2018 Apr 17;9:788. doi: 10.3389/fimmu.2018.00788 (PMC5913352; doi:10.3389/fimmu.2018.00788)

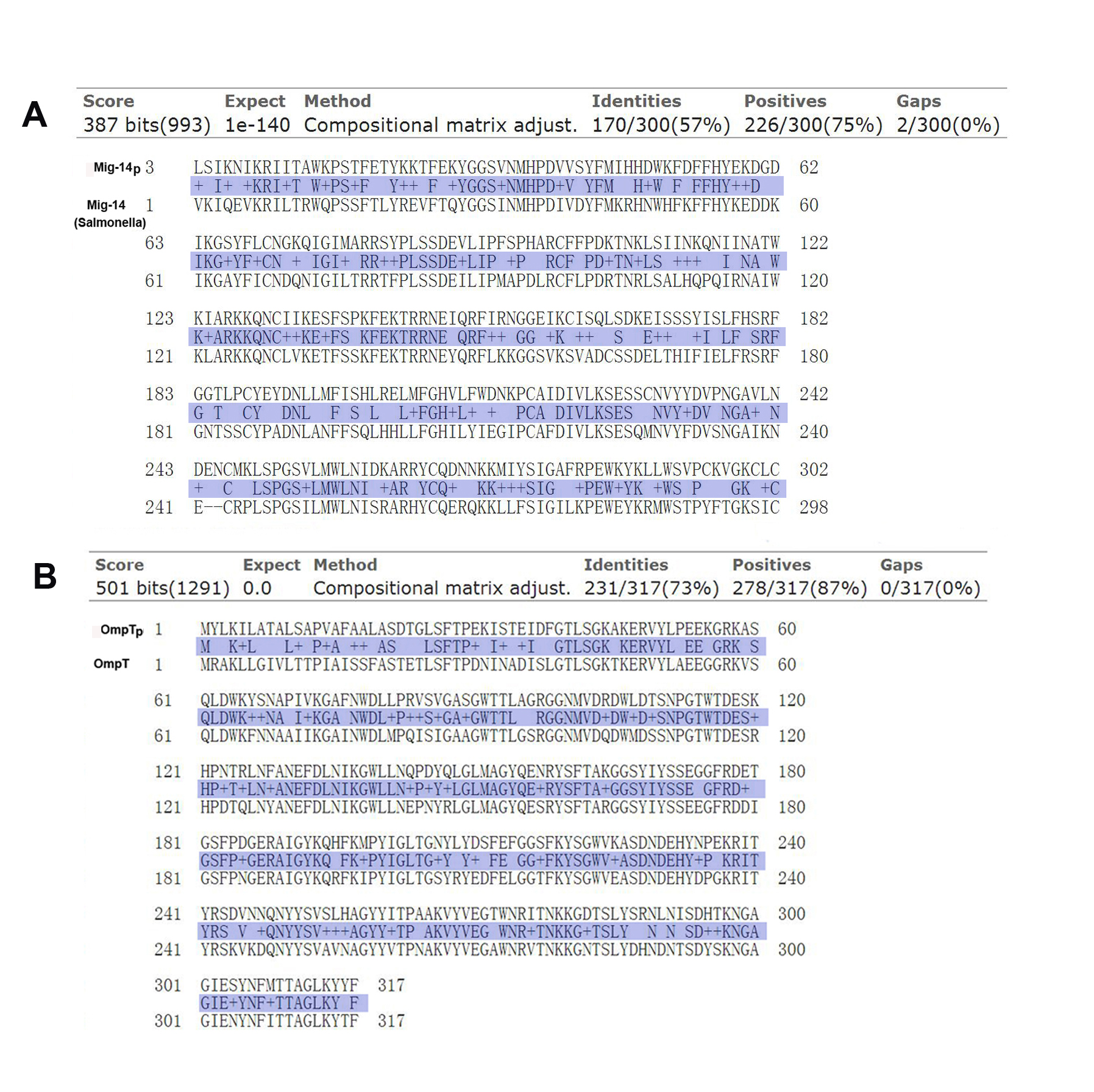

Supplement: Figure S1 — Sequence comparison of (A) Mig-14p and ortholog (Mig-14) in Salmonella and (B) alignment of OmpTp and the homolog of Escherichia coli chromosome-encoding OmpT. [file Image_1.TIF]

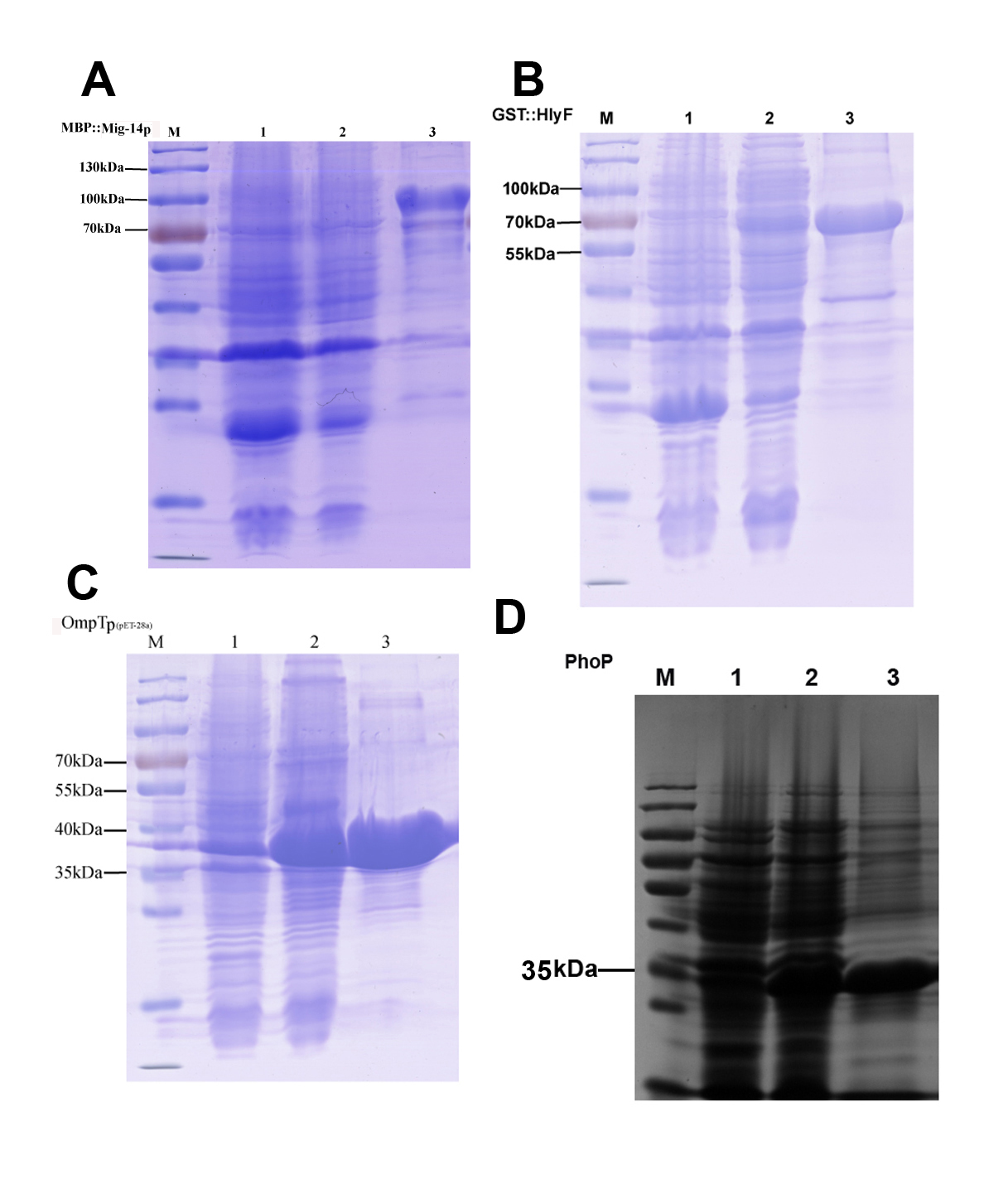

Supplement: Figure S2 — Purification of MBP::Mig-14p (A), GST::HlyF (B), OmpTp (C), and PhoP (D) fusion proteins expressed in Escherichia coli BL21(DE3). Proteins from the total bacterial extracts (lanes 1 and 2) and the purified fusion proteins (lane 1) were separated on an SDS-PAGE with Coomassie, respectively. Lane M, protein marker. [file Image_2.TIF]

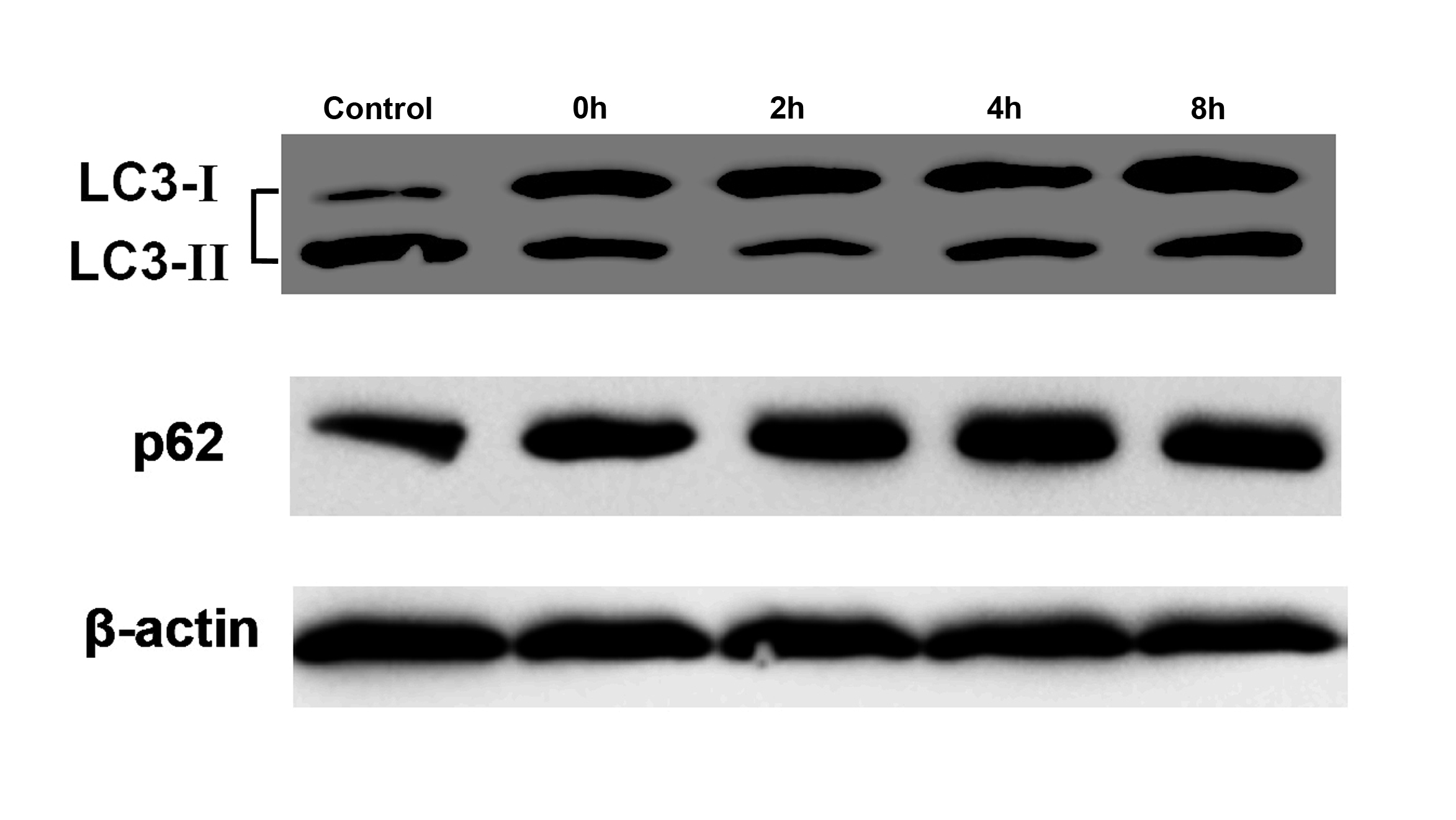

Supplement: Figure S3 — Western blots of LC3 and p62 in uninfected HD11 cells. HD11 cells were treated with rapamycin for 4 h as a positive control of autophagy induction, and incubated with fetal bovine serum-free RPMI 1640 media as the negative control. At 0, 2, 4, or 8 h post, the uninfected cells were lysed, and SDS-PAGE was performed. Western blot using antibodies against LC3, p62, and β-actin protein as indicated. [file Image_3.JPEG]

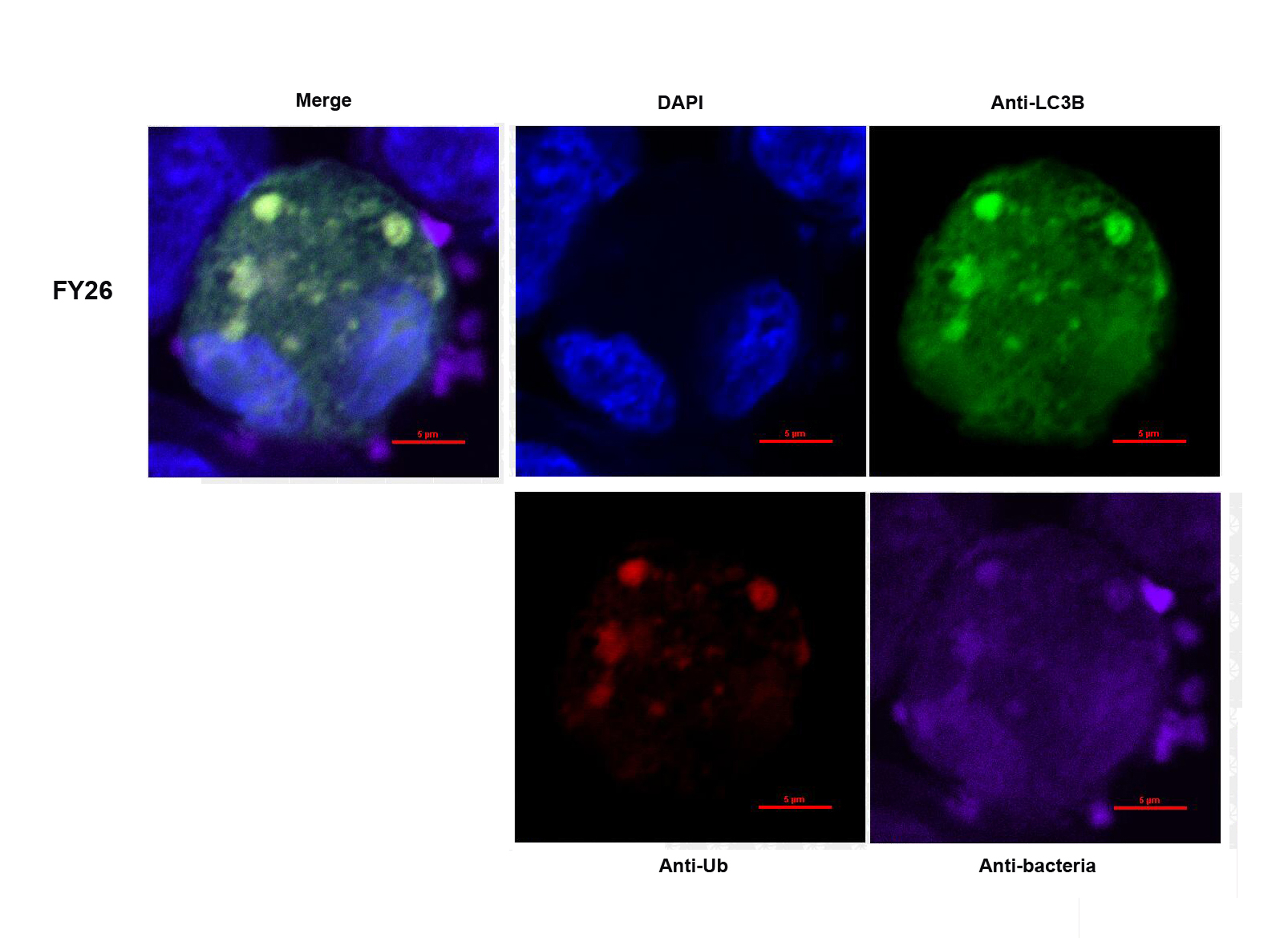

Supplement: Figure S4 — The immunofluorescence labeling was performed to identify the intracellular localization of LC3 and ubiiquitin (Ub) with wild-type FY26 at a multiplicity of infection of 5. Bacteria were labeled with anti-LC3 antibodies (FITC, green), anti-ExPEC antibody (Alexa 647, purple), and anti-Ub antibodies (TRITC, red). DNA was dyed with DAPI (blue). Representative confocal microscopy images for 4 hpi were shown. Scale bar = 5 µm. [file Image_4.TIF]
