# Supplementary material for: A Novel PhoP/PhoQ Regulation Pathway Modulates the Survival of Extraintestinal Pathogenic Escherichia coli in Macrophages
Source: Front Immunol. 2018 Apr 17;9:788. doi: 10.3389/fimmu.2018.00788 (PMC5913352; doi:10.3389/fimmu.2018.00788)
Supplement: Table S1 — Bacterial strains and plasmids used in this study. [file Table_1.DOCX]

**Table S1 Bacterial strains and plasmids used in this study.**

| **Bacterial strains and plasmids** | **Genotype or relevant characteristics** | **Source or Reference** |
| --- | --- | --- |
| **Bacterial strains** |  |  |
| *E. coli* DH5α | Plasmid propagation strain | Invitrogen |
| *E. coli* BL21 (DE3) | F-, *ompT, hsdS (r_B_^-^ m_B_^-^) gal, dcm* (DE3) | TIANGEN |
| FY26 | O2:K1; ST 95; phylogroup B2 | ([1-3](#_ENREF_1)) |
| FY26ΔHlyF | *hlyF* deletion in FY26 | This study |
| FY26ΔMig-14p | *Mig-14p* deletion in FY26 | This study |
| FY26ΔOmpTp | *OmpTp* deletion in FY26 | This study |
| FY26ΔPhoP | *phoP* deletion in FY26 | This study |
| FY26ΔOmpT | *ompT* deletion in FY26 | This study |
| FY26ΔOmpTp/OmpT | Parallel deletion of *OmpTp* and *ompT* in FY26 | This study |
| FY26ΔHlyF/pMig-14 | Parallel deletion of *hlyF* and *Mig-14p* in FY26 | This study |
| FY26ΔMig-14p | Deletion of *pMig-14* in FY26 | This study |
| FY26CHlyF/pMig-14 | FY26ΔHlyF/Mig-14p with plasmid pSTV28-*hlyF*/*Mig-14p* | This study |
| FY26CPhoP | FY26ΔPhoP with plasmid pSTV28-*phoP* | This study |
| FY26CHlyF | FY26ΔHlyF with plasmid pSTV28-*hlyF* | This study |
| FY26COmpTp | FY26ΔOmpTp with plasmid pSTV28-*OmpTp* | This study |
| RS218 | The prototypic NMEC strain without ColV plasmids | ([4](#_ENREF_4)) |
| RS218CHlyF | The complemented RS218 variant with plasmid pSTV28-*hlyF* | This study |
| RS218CHlyF/Mig-14p | The complemented RS218 variant with pSTV28-*hlyF*/*Mig-14p* | This study |
| RS218COmpTp | The complemented RS218 variant with plasmid pSTV28-*OmpTp* | This study |
| FY26Δ*lacI-Z* | Parallel deletion of *lacI* and *lacZ* in FY26 | This study |
| FY26Δ*lacI-Z hlyF*::*lacZ-zeo* | The *hlyF::lacZ-zeo* transcriptional reporter fusion in FY26Δ*lacI-Z* | This study |
| FY26Δ*lacI-Z/phoP hlyF*::*lacZ-zeo* | *phoP* deletion in FY26Δ*lacI-Z upaB::lacZ-zeo* | This study |
| FY26Δ*lacI-Z-*CphoP *hlyF::lacZ-zeo* | The complementary pSTV28-*phoP* in FY26Δ*lacI-Z upaB::lacZ-zeo* | This study |
|  |  |  |
| ***Plasmids*** |  |  |
| pCold-*malE* | fusing *malE* coding sequence into the pColdⅠ(TaKaRa) | ([5](#_ENREF_5)) |
| pCold-*malE*/*pMig-14* | pCold-*malE* carrying *Mig-14p* gene | This study |
| pCold-GST | fusing GST coding sequence into the pColdⅠ(TaKaRa) | This study |
| pCold-*GST*/*hlyF* | pCold-*GST* carrying *hlyF* gene | This study |
| pET-28a(+) | Prokaryotic expression plasmid | Novagen |
| pET-28a-*OmpTp* | pET-28a carrying *OmpTp* gene | This study |
| pET-28a-*phoP* | pET-28a carrying *phoP* gene | This study |
| pSTV28 | A medium-copy plasmid | Takara |
| pSTV28-*hlyF* | pSTV28 carrying *hlyF* coding region and its putative promoter | This study |
| pSTV28-*hlyF*/*Mig-14p* | pSTV28 carrying *hlyF* and *pMig-14* coding regions and its putative promoter | This study |
| pSTV28-*OmpTp* | pSTV28 carrying *OmpTp* coding region and its putative promoter | This study |
| pSTV28-*phoP* | pSTV28 carrying *phoP* coding region and its putative promoter | This study |
| pKD4 | template for λ-Red Kan^r^ cassette | ([6](#_ENREF_6)) |
| pCP20 | encodes FLP recombinase for removal of resistance cassette | ([6](#_ENREF_6)) |
| pKD46 | λ-Red recombinase expression | ([6](#_ENREF_6)) |

**References**

1. Zhu-Ge XK, Pan ZH, Tang F, Mao X, Hu L, Wang SH, et al. The effects of *upaB* deletion and the double/triple deletion of *upaB*, *aatA*, and *aatB* genes on pathogenicity of avian pathogenic *Escherichia coli*. *Appl Microbiol Biotechnol* (2015). Epub 2015/08/19. doi: 10.1007/s00253-015-6925-2. PubMed PMID: 26278540.

2. Zhu Ge X, Jiang J, Pan Z, Hu L, Wang S, Wang H, et al. Comparative genomic analysis shows that avian pathogenic *Escherichia coli* isolate IMT5155 (O2:K1:H5; ST complex 95, ST140) shares close relationship with ST95 APEC O1:K1 and human ExPEC O18:K1 strains. *PLoS One* (2014) 9(11):e112048. Epub 2014/11/15. doi: 10.1371/journal.pone.0112048 PONE-D-14-12415 [pii]. PubMed PMID: 25397580; PubMed Central PMCID: PMC4232414.

3. Zhuge X, Wang S, Fan H, Pan Z, Ren J, Yi L, et al. Characterization and functional analysis of AatB, a novel autotransporter adhesin and virulence factor of avian pathogenic *Escherichia coli*. *Infect Immun* (2013) 81(7):2437-47. Epub 2013/05/01. doi: 10.1128/IAI.00102-13 [pii]. PubMed PMID: 23630958; PubMed Central PMCID: PMC3697619.

4. Mittal R, Prasadarao NV. gp96 expression in neutrophils is critical for the onset of *Escherichia coli* K1 (RS218) meningitis. *Nat Commun* (2011) 2:552. Epub 2011/11/24. doi: 10.1038/ncomms1554 [pii]. PubMed PMID: 22109526; PubMed Central PMCID: PMC3537828.

5. Zhuge X, Tang F, Zhu H, Mao X, Wang S, Wu Z, et al. AutA and AutR, Two Novel Global Transcriptional Regulators, Facilitate Avian Pathogenic *Escherichia coli* Infection. *Sci Rep* (2016) 6:25085. Epub 2016/04/27. doi: 10.1038/srep25085[pii]. PubMed PMID: 27113849; PubMed Central PMCID: PMC4844996.

6. Datsenko KA, Wanner BL. One-step inactivation of chromosomal genes in *Escherichia coli* K-12 using PCR products. *Proc Natl Acad Sci U S A* (2000) 97(12):6640-5. Epub 2000/06/01. doi: 10.1073/pnas.120163297[pii]. PubMed PMID: 10829079; PubMed Central PMCID: PMC18686.
