# Supplementary material for: A Novel PhoP/PhoQ Regulation Pathway Modulates the Survival of Extraintestinal Pathogenic Escherichia coli in Macrophages
Source: Front Immunol. 2018 Apr 17;9:788. doi: 10.3389/fimmu.2018.00788 (PMC5913352; doi:10.3389/fimmu.2018.00788)
Supplement: Table S2 — Oligonucleotide sequences used as PCR primers in this study. [file Table_2.docx]

Table S2. Oligonucleotide sequences used as PCR primers.

| **Primers** | **Sequence (5'-3')** |
| --- | --- |
| **General PCR for cloning** |  |
| pSTV28-*hlyF*-F | CGAGCTCCCAGCACTTCTGATGGTGGTCTT |
| pSTV28-*hlyF*-R | CCCAAGCTTTTTAAAATCAACTTCCATTTGTTGTT |
| pSTV28-*hlyF*/*Mig-14p*-F | CGAGCTCCCAGCACTTCTGATGGTGGTCTT |
| pSTV28-*hlyF*/*Mig-14p*-R | CCCAAGCTTGCCCTGACTCAAAGAAAGAAATG |
| pSTV28-*OmpTp*-F | CGAGCTCTAACAAAATAAACCCAGGAAGAAGG |
| pSTV28-*OmpTp*-R | CCCAAGCTTACACTGTGTCGTTATGCATGAAGAG |
| pSTV28-*phoP*-F | CGAGCTCCTAACTACATTGGTCGCGCCATC |
| pSTV28-*phoP*-R | CCCAAGCTTCCGATCAGCGCAACCATTCCGTAG |
| pCold-*malE*/*Mig-14p*-F | CCGCTCGAGATGACTTTGTCAATTAAAAATATAAAGCG |
| pCold-*malE*/*Mig-14p*-R | CCGGAATTCACATAAGCATTTACCAACCTTACAGG |
| pCold-*GST*/*hlyF*-F | CCGCTCGAGATGAAATTGTTATTACTTACAGGTGCA |
| pCold-*GST*/*hlyF*-R | CCGGAATTCTTTAAAATCAACTTCCATTTGTTGTT |
| pET-28a-*OmpTp*-F | CGAGCTCTCTGATACTGGGCTTTCTTTTACACC |
| pET-28a-*OmpTp*-R | CCGCTCGAGAAAATAATACTTCAGACCAGCTGTGG |
| pET-28a-*phoP*-F | CCGGAATTCATGCGCGTACTGGTTGTTGA |
| pET-28a-*phoP*-R | CCGCTCGAGTCAGCGCAATTCGAACAGAT |
| ***For lacZ fusion*^a^** |  |
| *lacZ*-F | ATGATTACATGAAAACAAAAGAGGTTGGATATGAAATTGTTATTACTTACAGGTGCAACAactatgattacggattctctggc |
| *lacZ*-R | TTATTTTTGACACCAGACCA |
| pKD4-F | TGGTCTGGTGTCAAAAATAAGTGTAGGCTGGAGCTGCTTC |
| pKD4-R | CCTCGGCGTTTTTATATATTATGAGTGCAACACCAACAATAATTCTGATTATGATAAATAcatatgaatatcctccttag |
| Fusion-F | ATGATTACATGAAAACAAAAGAGG |
| Fusion-R | CCTCGGCGTTTTTATATATTATGAGTG |
| **For Deletion^a^** |  |
| Del-*hlyF*-F | TAAGATAATTTATTTTTATAATGATTACATGAAAACAAAAGAGGTTGGATgtgtaggctggagctgcttc |
| Del-*hlyF*-R | TTTATATATTATGAGTGCAACACCAACAATAATTCTGATTATGATAAATAcatatgaatatcctccttag |
| Del-*hlyF/Mig-14p*-F | TAAGATAATTTATTTTTATAATGATTACATGAAAACAAAAGAGGTTGGATgtgtaggctggagctgcttc |
| Del-*hlyF/Mig-14p*-R | GTATTTGCAGACTGGACGCTCTTATATAGTTACTTACCTTTATACATATTcatatgaatatcctccttag |
| Del-*lacI-Z*-F | AAAACCTTTCGCGGTATGGCATGATAGCGCCCGGAAGAGAGTCAATTCAGGGTGGTGAATgtgtaggctggagctgcttc |
| Del-*lacI-Z*-R | ATAGTACATAATGGATTTCCTTACGCGAAATACGGGCAGACATGGCCTGCCCGGTTATTAcatatgaatatcctccttag |
| Del-*OmpTp*-F | TACCGGTTTCCATGTTACAGACGTTCCCGGCATAAAGTGTCCGACCAGGGgtgtaggctggagctgcttc |
| Del-*OmpTp*-R | TTATCATATTTAAAGTGTCATGAAAAAATTAAAAACAGGCATGTTTCAGGcatatgaatatcctccttag |
| Del-*Mig-14p*-F | ATAACTTTGTCAATTAAAAATATAAAGCGCATTATTACGGCCTGGAAACCgtgtaggctggagctgcttc |
| Del-*Mig-14p*-R | GTATTTGCAGACTGGACGCTCTTATATAGTTACTTACCTTTATACATATTcatatgaatatcctccttag |
| Del-*phoP*-F | ATAACCACATAATCGCGTTACACTATTTTAATAATTAAGACAGGGAGAAATAAAAgtgtaggctggagctgcttc |
| Del-*phoP*-R | TATCTGCAACCGATTATAACGGATGCTTAACGTAATGCGTGAAGTATGGACATATcatatgaatatcctccttag |
| **For EMSA** |  |
| P*_hlyF_*-F | TCAATAATAAGCAGAACATCCAA |
| P*_hlyF_*-R | TGTTGCACCTGTAAGTAATAACAAT |
| *hlyF*-F | CAGGACTGAAACGCTTCCTTCATGT |
| *hlyF*-R | AATAATTGATGGTCGGGCAACCAA |
| **For co-transcription** |  |
| Opr-Transposase/*pOmpT*-F | ATATCAACCACCTGAGTTTATTTT |
| Opr-Transposase/*pOmpT*-R | AAACGCTACAGGAGCTGAGAGTGC |
| Opr-*OmpTp*/*hlyF*-F | GTGCAGGCATTGAAAGCTATAAC |
| Opr-*OmpTp*/*hlyF*-F | GAAATCCTGTTGCACCTGTAAGT |
| Opr-*hlyF*/*Mig-14p*-F | GCCTTATGTTAAAAGCCATTCGT |
| Opr-*hlyF*/*Mig-14p*-R | CGGATGCATGTTTACACTACCAC |
| Opr-Mig-14p/Recombinase-F | ATGATATATTCTATCGGTGCATTCA |
| Opr-Mig-14p/Recombinase-R | GCGGGCTGAGGGCTGGTTTACCGT |
| **For RT-PCR** |  |
| *hlyF*-qPCR-F | AGTGTGACGTTCTCTGCTATTG |
| *hlyF* -qPCR-R | CGGCTCATTGCCAGTATGT |
| *Mig-14p*-qPCR-F | CAGGAATGGCGGAGAGATTAAG |
| *Mig-14p*-qPCR-R | CCTCCAAACCGGGAATGAAA |
| *OmpTp*-qPCR-F | GGGTGAAGGCATCGGATAAT |
| *OmpTp*-qPCR-R | CTGTCTCACTACATGCTGGTT |
| *OmpT*-qPCR-F | GGGTGAAGGCATCGGATAAT |
| *OmpT*-qPCR-R | AACCAGCATGTAGTGAGACAG |
|  |  |
|  |  |

- Underlined are restriction cutting sites;
- Capital letters represent homologous fragments of the deleted genes.
