# Supplementary material for: A Novel PhoP/PhoQ Regulation Pathway Modulates the Survival of Extraintestinal Pathogenic Escherichia coli in Macrophages
Source: Front Immunol. 2018 Apr 17;9:788. doi: 10.3389/fimmu.2018.00788 (PMC5913352; doi:10.3389/fimmu.2018.00788)
Supplement: Table S3 — MICs of antimicrobial peptides (AMPs) (LL-37 and HBD2). [file Table_3.docx]

Table S3. MICs of AMPs (LL-37 and HBD2)

| Strain | MICs (μg/ml) of AMPs (LL-37 and HBD2) | |
| --- | --- | --- |
|  | LL-37 | HBD2 |
| FY26 | 30-60 | 30 |
| RS218 | 15 | 7.5 |
| FY26ΔMig-14p | 7.5 | 7.5 |
| FY26ΔHlyF | 30-60 | 30 |
| FY26ΔHlyF/Mig-14p | 7.5 | 7.5 |
| FY26CHlyF/Mig-14p | 60 | 60 |
| FY26ΔPhoP | 3.75 | 3.75 |
| FY26CPhoP | 30 | 15 |
| FY26ΔOmpTp | 15-30 | 15 |
| FY26ΔOmpT | 30 | 15-30 |
| FY26ΔOmpTp/OmpT | 15 | 15 |
| RS218CHlyF | 15 | 7.5 |
| RS218CHlyF/Mig-14p | 60 | 30-60 |
| RS218COmpTp | 30 | 15 |
